# Supplementary material for: Efficient generation of isogenic primary human myeloid cells using CRISPR-Cas9 ribonucleoproteins
Source: Cell Rep. Author manuscript; Available in PMC 2021 Jun 9. (PMC8188731; doi:10.1016/j.celrep.2021.109105)
Supplement: 1 [file NIHMS1709014-supplement-1.pdf]

Cell Reports, Volume 35

Supplemental information

Efficient generation of isogenic primary human myeloid cells using CRISPR-Cas9 ribonucleoproteins

Joseph Hiatt, Devin A. Cavero, Michael J. McGregor, Weihao Zheng, Jonathan M. Budzik, Theodore L. Roth, Kelsey M. Haas, David Wu, Ujjwal Rathore, Anke Meyer-Franke, Mohamed S. Bouzidi, Eric Shifrut, Youjin Lee, Vigneshwari Easwar Kumar, Eric V. Dang, David E. Gordon, Jason A. Wojcechowskyj, Judd F. Hultquist, Krystal A. Fontaine, Satish K. Pillai, Jeffery S. Cox, Joel D. Ernst, Nevan J. Krogan, and Alexander Marson

Figure S1

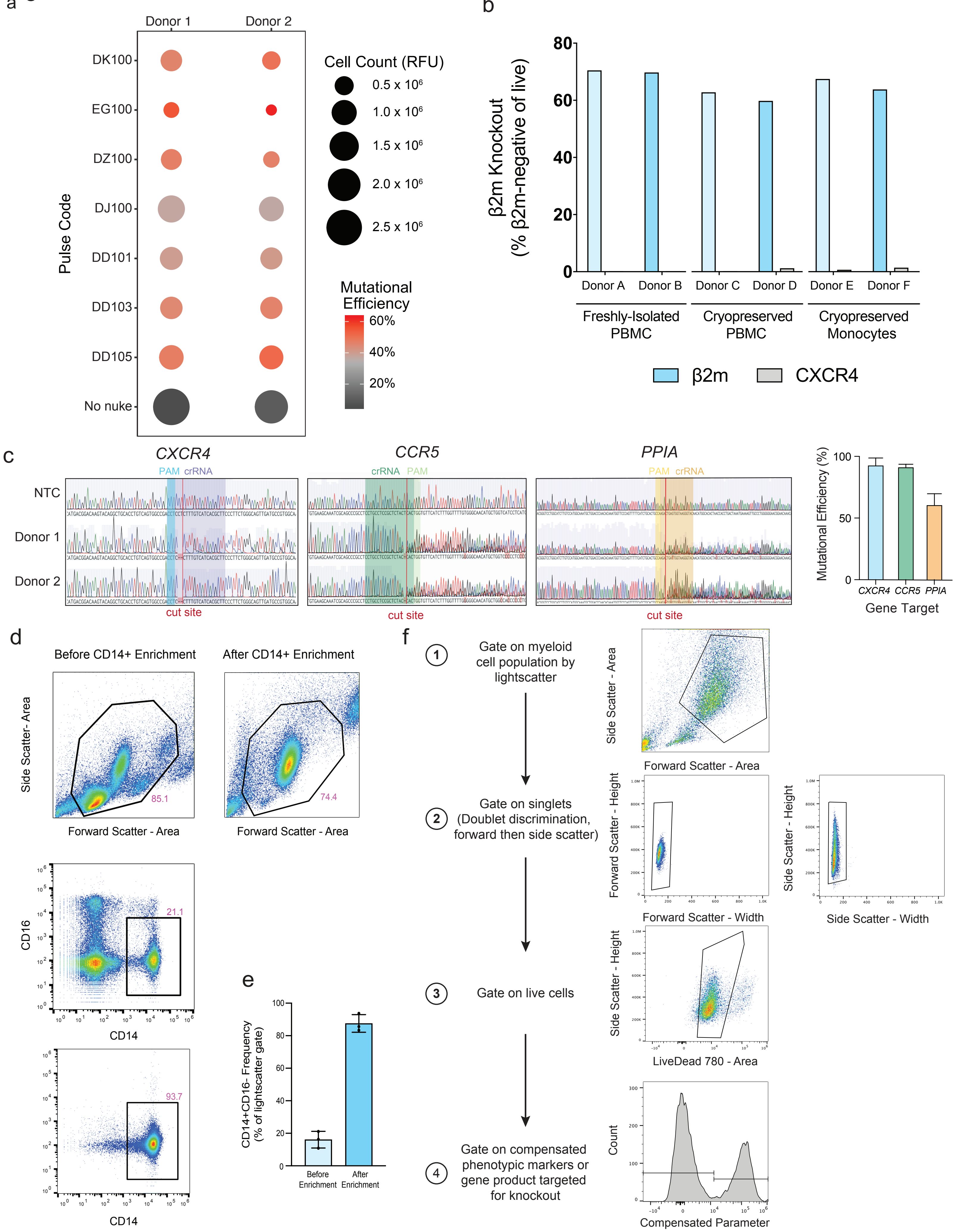
a
Donor 1
Donor 2
DK100
EG100
DZ100
DJ100
DD101
DD103
DD105
No nuke
Pulse Code
Cell Count (RFU)
0.5 x 10^6
1.0 x 10^6
1.5 x 10^6
2.0 x 10^6
2.5 x 10^6
Mutational Efficiency
60%
40%
20%
b
β2m Knockout (% β2m-negative of live)
80
60
40
20
0
Donor A
Donor B
Donor C
Donor D
Donor E
Donor F
Freshly-Isolated PBMC
Cryopreserved PBMC
Cryopreserved Monocytes
β2m
CXCR4
c
CXCR4
CCR5
PPIA
PAM
crRNA
NTC
Donor 1
Donor 2
cut site
Mutational Efficiency (%)
100
50
0
CXCR4
CCR5
PPIA
Gene Target
d
Before CD14+ Enrichment
After CD14+ Enrichment
Side Scatter- Area
Forward Scatter - Area
85.1
74.4
CD16
CD14
21.1
93.7
e
CD14+CD16- Frequency (% of lightscatter gate)
100
80
60
40
20
0
Before Enrichment
After Enrichment
f
1 Gate on myeloid cell population by lightscatter
2 Gate on singlets (Doublet discrimination, forward then side scatter)
3 Gate on live cells
4 Gate on compensated phenotypic markers or gene product targeted for knockout
Side Scatter - Area
Forward Scatter - Area
Forward Scatter - Height
Forward Scatter - Width
Side Scatter - Height
Side Scatter - Width
LiveDead 780 - Area
Count
Compensated Parameter

Figure S1. Optimization of knockout in fresh and cryopreserved CD14+ monocytes and notes on isolation, Related to Figure 1 and STAR Methods. (a) Lonza nucleofection pulse code optimization of *SAMHD1* knockout using guide SAMHD1-2 in CD14+ monocytes freshly isolated from blood of two healthy donors. All nucleofections occurred in buffer P2. Color indicates mutational efficiency determined by TIDE, size of circles indicates relative surviving cell count measured by CellTiter-Glo fluorescent assay in relative fluorescence units (RFU), mean of technical triplicates. Cells were also visually monitored for health and morphology. **(b)** Knockout efficiency in CD14+ monocytes freshly isolated from blood (Donors A and B), CD14+ monocytes freshly isolated from cryopreserved PBMC (Donors C and D), and cryopreserved CD14+ monocytes (Donors E and F). Knockout at the targeted *β2m* locus was determined by TIDE compared to the non-targeting control (blue bars, left side of each donor); grey bars represent the TIDE knockout efficiency at the *β2m* locus of off-target *CXCR4* RNPs (grey bars, right side of each donor.) **(c)** Genomic analysis of knockout target sites allows for quantification of mutational efficiency independent of gene product expression. Left, representative chromatograms of non-targeting control (top) and edited (middle, Donor 1; bottom, Donor 2) sequences, with crRNA sequence, cut site and protospacer-adjacent motif (PAM) highlighted; right, quantification of editing efficiency by TIDE. Bars represent mean ± SD of two (*PPIA*) or four (*CXCR4, CCR5*) biological replicates. **(d)** Representative lightscatter (top) and CD14 vs. CD16 staining (bottom) of density-separated PBMC (left) and negatively selected CD14+ monocytes (right). **(e)** Quantification of enrichment by magnetic negative selection across three donors. Bars represent mean ± SD with individual points marked. **(f)** Panels illustrating four-step sample gating strategy for myeloid cells.

Figure S2

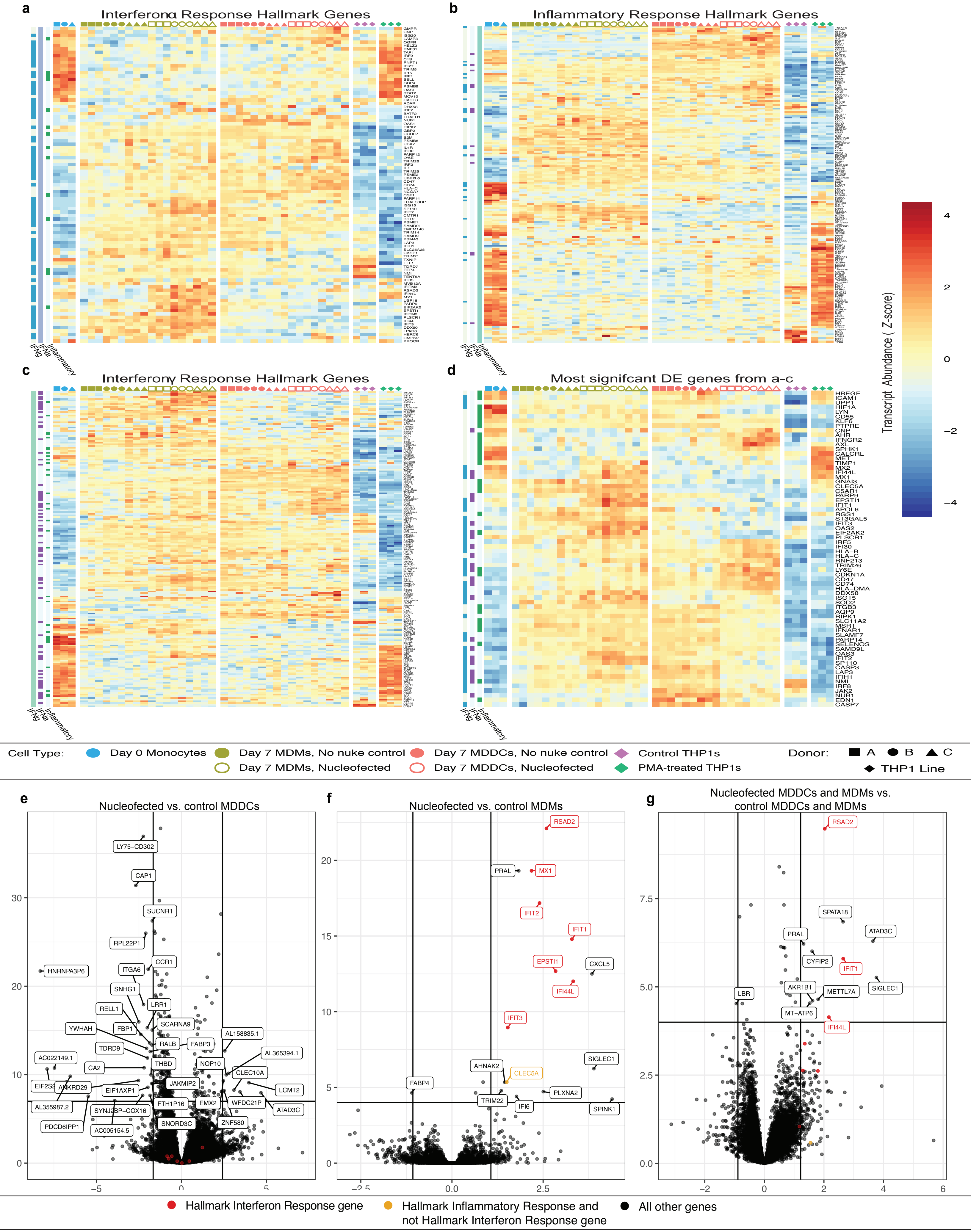
a
Interferonα Response Hallmark Genes
b
Inflammatory Response Hallmark Genes
c
Interferonγ Response Hallmark Genes
d
Most signifcant DE genes from a-c
IFNg
IFNa
Inflammatory
HBEGF
ICAM1
UPP1
HIF1A
LYN
CD55
KLF6
PTPRE
CNP
AHR
IFNGR2
AXL
SPHK1
CALCRL
MET
TIMP1
MX2
IFI44L
MX1
GNAI3
CLEC5A
C5AR1
PARP9
EPSTI1
IFIT1
APOL6
RGS1
ST3GAL5
IFIT3
OAS2
EIF2AK2
PLSCR1
IRF5
IFI30
HLA-B
HLA-C
RNF213
TRIM26
LY6E
CDKN1A
CD47
CD74
HLA-DMA
DDX58
ISG15
SOD2
ITGB3
AQP9
RIPK1
SLC11A2
MSR1
IFNAR1
SLAMF7
PARP14
SELENOS
SAMD9L
OAS3
IFIT2
SP110
CASP3
LAP3
IFIH1
NMI
IRF8
JAK2
NUB1
EDN1
CASP7
Transcript Abundance (Z-score)
4
2
0
−2
−4
Cell Type:
Day 0 Monocytes
Day 7 MDMs, No nuke control
Day 7 MDMs, Nucleofected
Day 7 MDDCs, No nuke control
Day 7 MDDCs, Nucleofected
Control THP1s
PMA-treated THP1s
Donor:
A
B
C
THP1 Line
e
Nucleofected vs. control MDDCs
LY75−CD302
CAP1
SUCNR1
RPL22P1
CCR1
ITGA6
HNRNPA3P6
SNHG1
LRR1
RELL1
SCARNA9
YWHAH
FBP1
AL158835.1
RALB
FABP3
TDRD9
AL365394.1
AC022149.1
THBD
NOP10
CA2
CLEC10A
JAKMIP2
EIF2S2
ANKRD29
EIF1AXP1
LCMT2
EMX2
WFDC21P
FTH1P16
AL355987.2
SYNJ2BP−COX16
ATAD3C
ZNF580
SNORD3C
PDCD6IPP1
AC005154.5
30
20
10
0
−5
0
5
f
Nucleofected vs. control MDMs
RSAD2
PRAL
MX1
IFIT2
IFIT1
EPSTI1
CXCL5
IFI44L
IFIT3
SIGLEC1
AHNAK2
CLEC5A
FABP4
PLXNA2
TRIM22
IFI6
SPINK1
20
15
10
5
0
−2.5
0.0
2.5
g
Nucleofected MDDCs and MDMs vs.
control MDDCs and MDMs
RSAD2
SPATA18
ATAD3C
PRAL
CYFIP2
IFIT1
SIGLEC1
LBR
AKR1B1
METTL7A
MT−ATP6
IFI44L
7.5
5.0
2.5
0.0
−2
0
2
4
6
Hallmark Interferon Response gene
Hallmark Inflammatory Response and not Hallmark Interferon Response gene
All other genes

Figure S2. Selected transcript abundance from monocytes, THP1 cells, and nucleofected and control MDMs and MDDCs, and differentially expressed transcripts, Related to Figure 2. (a-d) Relative transcript abundance for transcripts in the **(a)** Hallmark Interferon Alpha Response gene set**, (b)** Hallmark Interferon Gamma Response gene set or **(c)** Hallmark Inflammatory Response gene set (Liberzon et al., 2015). As the three sets are overlapping, the set memberships for each gene are displayed to the left of each heatmap. **(d)** Relative transcript abundance for leading edge genes from **(a-c)** with adjusted *P* value < 0.01 that drive differences between nucleofected and unperturbed MDMs or MDDCs. **(e-g)** Volcano plot of **(e)** nucleofected versus control day 7 MDDCs, **(f)** nucleofected versus control day 7 MDMs, **(g)** and combined nucleofected versus control day 6 MDDCs and MDMs. Transcripts in the top and bottom 1% by $\log_2$ fold change (x axis, cutoffs depicted by vertical lines) with adjusted *P* values less than 1×10^{-7} (panel **e**) or 1×10^{-4} (panels **f,g**) are labeled. Red dots indicate genes in the Hallmark Interferon Alpha Response or Hallmark Interferon Gamma Response gene sets (Liberzon et al., 2015); orange dots indicate genes not in the above two gene sets within the Hallmark Inflammatory Response gene set.

Figure S3

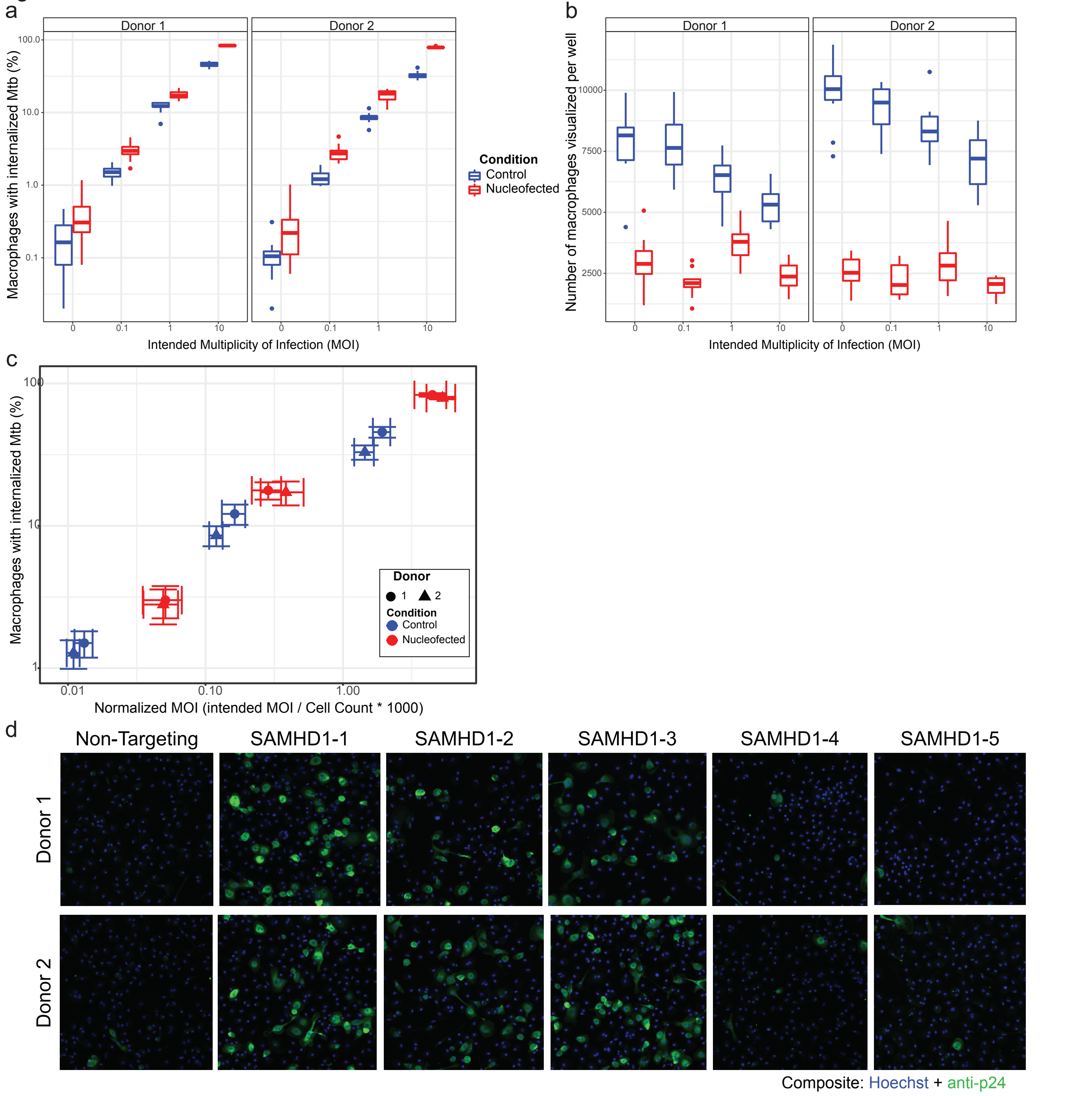

e

Dunnett's Multiple Comparison	Mean Difference (% infection)	95% CI of difference	Adjusted P Value
Non-Targeting vs. SAMHD1-1	21.05%	6.017% – 36.09%	0.0199
Non-Targeting vs. SAMHD1-2	20.55%	11.31% – 29.79%	0.0053
Non-Targeting vs. SAMHD1-3	19.08%	-0.5983% – 38.76%	0.0543
Non-Targeting vs. SAMHD1-4	1.10%	-0.4300% – 2.623%	0.1156
Non-Targeting vs. SAMHD1-5	0.82%	-0.5857% – 2.234%	0.1849

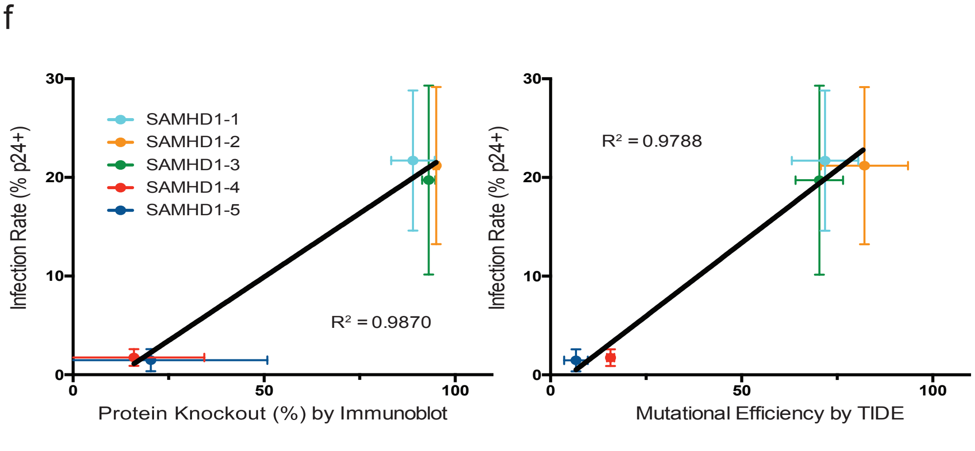

Figure S3. Quantification of phagocytosis of *Mycobacterium tuberculosis* and infection by HIV-1, Related to Figures 2-3.
(a-c) Quantification of phagocytosis of *Mycobacterium tuberculosis* (*Mtb*) by nucleofected and unperturbed day 7 monocyte-derived macrophages (MDMs). **(a)** Percentage of macrophages with internalized *Mtb* by intended multiplicity of infection (MOI). Box and whisker plots summarize 12 technical replicates, where the box depicts mean and interquartile range with max and min, excluding outliers, represented by the end of the whiskers. **(b)** Number of macrophages per well by intended multiplicity of infection (MOI). Box and whisker plots summarize 12 technical replicates, where the box depicts mean and interquartile range with max and min, excluding outliers, represented by the end of the whiskers. **(c)** Percentage of macrophages with internalized *Mtb* by normalized MOI (normalized MOI = intended MOI / cell count in thousands). Error bars represent standard deviation. **(d-f)** Knockout of the host restriction factor SAMHD1 leads to increases in HIV infection in a manner correlated with guide efficiency. **(d)** Representative composite images of HIV-1 infection from Donors 1 and 2 comparing cells nucleofected with RNPs made from each *SAMHD1*-targeting crRNA and cells nucleofected with control non-targeting RNPs. Blue, Hoechst; green, staining of the HIV-1 antigen p24. **(e)** Statistical comparison of infection rates (percent of cells staining positive for HIV-1 antigen p24). All values were generated by GraphPad Prism using a repeated measures one-way ANOVA followed by Dunnett's multiple comparison test. **(f)** Correlation of HIV-1 infection rate and knockout efficiency as measured by immunoblot (left panel) or mutational efficiency as measured by Sanger sequencing quantified by TIDE (right panel). Points represent mean across four donors, except for immunoblot of guide 1 (n = 2) and TIDE of guides 1, 3 and 4 (n = 3); error bars represent standard deviation. Line of best fit and R^2 values were generated by linear regression in GraphPad Prism.
