# Supplementary material for: Efficient generation of isogenic primary human myeloid cells using CRISPR-Cas9 ribonucleoproteins
Source: Cell Rep. Author manuscript; Available in PMC 2021 Jun 9. (PMC8188731; doi:10.1016/j.celrep.2021.109105)
Supplement: 4 [file NIHMS1709014-supplement-4.pdf]

Cell Reports

Report

Efficient generation of isogenic primary human myeloid cells using CRISPR-Cas9 ribonucleoproteins

Graphical abstract

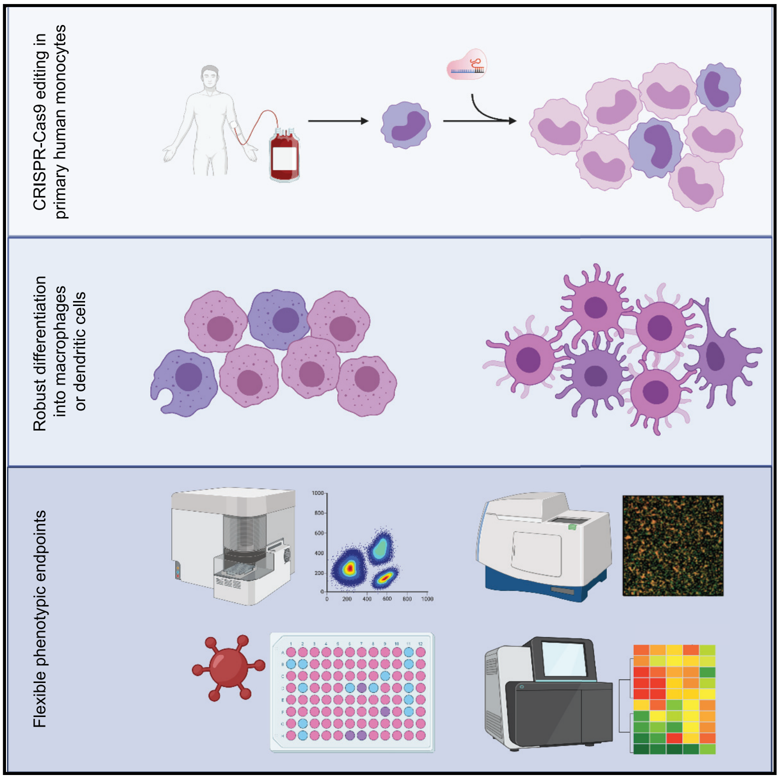

Highlights

- Cas9 ribonucleoproteins (RNPs) allow robust gene knockout in primary human monocytes
- Cas9 RNP nucleofected monocytes can differentiate into macrophages or dendritic cells
- Cas9 RNP nucleofected macrophages appropriately phagocytose *M. tuberculosis*
- Knockout of the host factor SAMHD1 increased HIV infection of macrophages >50×

Authors

Joseph Hiatt, Devin A. Cavero, Michael J. McGregor, ..., Joel D. Ernst, Nevan J. Krogan, Alexander Marson

Correspondence

nevan.krogan@ucsf.edu (N.J.K.), alexander.marson@ucsf.edu (A.M.)

In brief

Hiatt et al. report a method for genome editing in primary human monocytes using CRISPR-Cas9 ribonucleoproteins (RNPs). These cells can be differentiated into macrophages or dendritic cells for downstream phenotypic assays. They demonstrate the value for functional host-pathogen studies through knockout of the HIV-1 restriction factor SAMHD1.

Hiatt et al., 2021, Cell Reports *35*, 109105
May 11, 2021 © 2021 The Authors.
https://doi.org/10.1016/j.celrep.2021.109105

CellPress

Cell Reports

CellPress
OPEN ACCESS

Report

Efficient generation of isogenic primary human myeloid cells using CRISPR-Cas9 ribonucleoproteins

Joseph Hiatt,[1,2,3,4,5,19] Devin A. Cavero,[3,4,5,19] Michael J. McGregor,[3,6,7] Weihao Zheng,[8] Jonathan M. Budzik,[9,10] Theodore L. Roth,[1,2,3,4,5] Kelsey M. Haas,[3,6,7] David Wu,[1,2] Ujjwal Rathore,[3,4,5] Anke Meyer-Franke,[3] Mohamed S. Bouzidi,[11,12] Eric Shifrut,[3,4,5] Youjin Lee,[3,4,5] Vigneshwari Easwar Kumar,[3,4,5] Eric V. Dang,[13] David E. Gordon,[3,6,7] Jason A. Wojcechowskyj,[3,6,7] Judd F. Hultquist,[3,6,7,14] Krystal A. Fontaine,[3] Satish K. Pillai,[11,12] Jeffery S. Cox,[9] Joel D. Ernst,[8] Nevan J. Krogan,[3,6,7,*] and Alexander Marson[3,4,5,10,15,16,17,18,20,*]

[1]Medical Scientist Training Program, University of California, San Francisco, San Francisco, CA 94143, USA
[2]Biomedical Sciences Graduate Program, University of California, San Francisco, San Francisco, CA 94143, USA
[3]J. David Gladstone Institutes, San Francisco, CA 94158, USA
[4]Department of Microbiology and Immunology, University of California, San Francisco, San Francisco, CA 94143, USA
[5]Innovative Genomics Institute, University of California, Berkeley, Berkeley, CA 94720, USA
[6]Department of Cellular and Molecular Pharmacology, University of California, San Francisco, San Francisco, CA 94158, USA
[7]Quantitative Biosciences Institute, QBI, University of California, San Francisco, San Francisco, CA 94158, USA
[8]Department of Medicine, Division of Experimental Medicine, University of California, San Francisco, San Francisco, CA 94143, USA
[9]Department of Molecular and Cell Biology, University of California, Berkeley, Berkeley, CA 94720, USA
[10]Department of Medicine, University of California, San Francisco, San Francisco, CA 94143, USA
[11]Vitalant Research Institute, San Francisco, CA 94118, USA
[12]Department of Laboratory Medicine, University of California, San Francisco, San Francisco, CA 94143, USA
[13]Department of Biochemistry and Biophysics, University of California, San Francisco, San Francisco, CA 94143, USA
[14]Division of Infectious Diseases, Feinberg School of Medicine, Northwestern University, Chicago, IL 60611, USA
[15]Diabetes Center, University of California, San Francisco, San Francisco, CA 94143, USA
[16]Parker Institute for Cancer Immunotherapy, San Francisco, CA 94129, USA
[17]UCSF Helen Diller Family Comprehensive Cancer Center, University of California, San Francisco, San Francisco, CA 94158, USA
[18]Chan Zuckerberg Biohub, San Francisco, CA 94158, USA
[19]These authors contributed equally
[20]Lead contact
*Correspondence: nevan.krogan@ucsf.edu (N.J.K.), alexander.marson@ucsf.edu (A.M.)
https://doi.org/10.1016/j.celrep.2021.109105

SUMMARY

Genome engineering of primary human cells with CRISPR-Cas9 has revolutionized experimental and therapeutic approaches to cell biology, but human myeloid-lineage cells have remained largely genetically intractable. We present a method for the delivery of CRISPR-Cas9 ribonucleoprotein (RNP) complexes by nucleofection directly into CD14$^+$ human monocytes purified from peripheral blood, leading to high rates of precise gene knockout. These cells can be efficiently differentiated into monocyte-derived macrophages or dendritic cells. This process yields genetically edited cells that retain transcript and protein markers of myeloid differentiation and phagocytic function. Genetic ablation of the restriction factor SAMHD1 increased HIV-1 infection >50-fold, demonstrating the power of this system for genotype-phenotype interrogation. This fast, flexible, and scalable platform can be used for genetic studies of human myeloid cells in immune signaling, inflammation, cancer immunology, host-pathogen interactions, and beyond, and could facilitate the development of myeloid cellular therapies.

INTRODUCTION

Myeloid cells are key players in the immune system in health and disease (Germic et al., 2019; Lapenna et al., 2018; Worbs et al., 2017). Monocytes and macrophages function in the immediate arm of the innate immune system, responding to pathogens or tissue damage and helping to regulate and resolve inflammation in tissue. As professional antigen-presenting cells, dendritic cells (DCs) orchestrate the adaptive immune response. Myeloid cells play central roles in processes ranging from development and homeostatic regulation to pathogen response, autoinflammatory disease, fibrosis, and malignancy (Chao et al., 2020; Engblom et al., 2016; Medzhitov and Janeway, 1997, 2000; Wynn et al., 2013). Improved understanding of the normal and pathogenic behaviors of these cells is crucial to furthering our mechanistic understanding of a broad range of disorders, offering hope for the discovery and advancement of new treatments.

Our ability to identify new therapeutic targets and construct novel cellular interventions has advanced in lockstep with our ability to genetically manipulate relevant primary cell types. For

This is an open access article under the CC BY license (http://creativecommons.org/licenses/by/4.0/).

example, mouse genetic approaches have exposed the remarkable diversity of mouse macrophages, and genetic ablation of myeloid subsets has paved the way for the therapeutic targeting of analogous cells in the clinic (Wynn et al., 2013). CRISPR-Cas9-mediated gene targeting has significantly expanded the potential of once-intractable cell types, facilitating important discovery efforts and enhanced cell therapy approaches in primary T cells (Roth et al., 2018; Schumann et al., 2015; Simeonov and Marson, 2019; Stadtmauer et al., 2020), as well as cures for debilitating genetic diseases using edited hematopoietic stem/progenitor cells (Foss et al., 2019; Wu et al., 2019).

Until now, CRISPR-Cas9 has been inefficient in primary human myeloid cells, limiting functional genetic studies and genome engineering in these key cells of the human immune system. The identification of SAMHD1 as the key restriction factor in myeloid cells that prevents efficient lentiviral transduction (Hrecka et al., 2011; Laguette et al., 2011) led directly to improved approaches for studying innate immunity and has been leveraged to generate more effective DC vaccines (Norton et al., 2015; Sunseri et al., 2011). Even so, studies of human myeloid cells continue to suffer from the difficulty of accessing and manipulating relevant cell subsets (Lee et al., 2018). Expanding the genetic toolkit with CRISPR-Cas9 would enable further dissection of the genetic circuits underlying the behavior and development of this remarkably diverse class of cells (Geissmann and Mass, 2015; Hancock et al., 2013), offering new insight and more specific, sophisticated targets for therapeutic manipulation.

We report here a robust, flexible, and cost-efficient platform for genetically modifying primary human $CD14^+$ monocytes, which can then be quickly differentiated into monocyte-derived macrophages (MDMs) or monocyte-derived DCs (MDDCs). We demonstrate the utility of using this system to study host-pathogen interactions; however, this approach is equally suited to the study of any number of other phenotypic outcomes. The platform is designed to be scalable and is compatible with workflows assessed by microscopy, flow cytometry, and a wide range of other common assays, and is suitable for the interrogation of both cell-intrinsic and non-cell-autonomous behaviors.

RESULTS

Efficient gene ablation in primary myeloid cells

$CD14^+$ monocytes are abundant in peripheral blood, representing ~10% of circulating leukocytes (Auffray et al., 2009), and can be differentiated *ex vivo* into MDMs or MDDCs (Figueroa et al., 2016; Jin and Kruth, 2016), making them the ideal starting point for the generation of isogenic primary myeloid cells. Monocytes are isolated from donor blood and immediately subjected to CRISPR-Cas9 ribonucleoprotein (RNP) nucleofection. They are then put into culture, allowing several days for turnover of the targeted gene product under conditions leading to differentiation into MDMs or MDDCs, after which the cells can be subjected to a range of functional, genotypic, and phenotypic assays (Figure 1A). A survey of conditions for the Lonza 4D Nucleofector identified pulse code DK-100 in buffer P2 as optimally balancing editing efficiency, cell morphology, and cell survival, with 35% the cell count at day 7 compared to unperturbed control samples (Figure S1A). Using this approach, we showed robust, guide-sequence-dependent knockout of genes expressed in myeloid cells by immunoblot of the gene product at day 7 of differentiation (Figure 1B). By testing multiple guides, we were able to reproducibly achieve at least a 75% reduction in targeted proteins GNE1 and ATP6V1A—both known to play roles in viral replication in myeloid cells (Gordon et al., 2020; Han et al., 2018)—relative to untargeted housekeeping control gene products (Figure 1C). This protocol led to reproducible knockout when starting with $CD14^+$ monocytes from freshly isolated peripheral blood mononuclear cells (PBMCs), from cryopreserved PBMC, or from isolated-then-cryopreserved $CD14^+$ monocytes (Figure S1B), allowing for a flexible workflow and enabling iterative experiments on consistent biological samples. Furthermore, we confirmed consistently robust knockout across biological replicates at the genetic level by tracking of indels by decomposition (TIDE) analysis (Brinkman et al., 2014), showing that guides against *CXCR4* and *CCR5* led to disruption in >90% of alleles (Figure S1C). Collectively, these data demonstrate efficient knockout of targeted genes in primary human myeloid cells using CRISPR-Cas9 RNPs.

Edited $CD14^+$ monocytes differentiate robustly into MDDCs and MDMs

We sought to establish that Cas9-RNP nucleofected cells could differentiate into both MDMs and MDDCs. We isolated $CD14^+$ monocytes from the blood of 3 healthy human blood donors, and profiled the transcriptome of the fresh monocytes as well as gene-targeted and unperturbed control MDDCs and MDMs after 7 days in culture (Figure 1A). We used RNPs targeting the safe harbor locus *AAVS1* to assess the effect of nucleofection and double-strand break formation and repair independent of gene knockout. Principal-component analysis showed that both macrophage differentiation conditions (Iscove's modified Dulbecco's medium [IMDM] with 20% human male AB serum) and DC differentiation conditions (IMDM with 1% human male AB serum, 50 ng/mL interleukin-4 [IL-4] and 50 ng/mL granulocyte macrophage-colony-stimulating factor [GM-CSF]) successfully remodeled the monocyte transcriptional landscape in a manner that was consistent across donors, with distinct cell state clusters observed for each cell type (Figure 2A). Genome targeting did not appreciably alter the differentiation of MDMs in this analysis, while some separation of nucleofected and unperturbed MDDCs was observed. These differences, mainly on the second principal-component axis (15% of variance), remained much smaller than the differences between differentiation conditions. Interestingly, phorbol-12-myristate-13-acetate (PMA)-stimulated THP1 cells, which have been used as proxies for macrophages (Qin, 2012; Tsuchiya et al., 1980, 1982), clustered near fresh monocytes in the principal-component analysis. This transcriptomic analysis underscores that even with Cas9 RNP nucleofection, differentiated MDMs and MDDCs are distinct from each other and from immortalized THP1 cells.

We next confirmed that Cas9 RNP-nucleofected monocytes differentiated as expected into MDMs and MDDCs (Lehtonen et al., 2007). Day 7 MDMs exhibited a high expression of expected markers, including *CD14*, the high-affinity Fc receptor gene *FCGR1A* (or *CD64*) and the complement component *C3* (Figure 2B). Day 7 MDDCs had lower levels of these transcripts, and

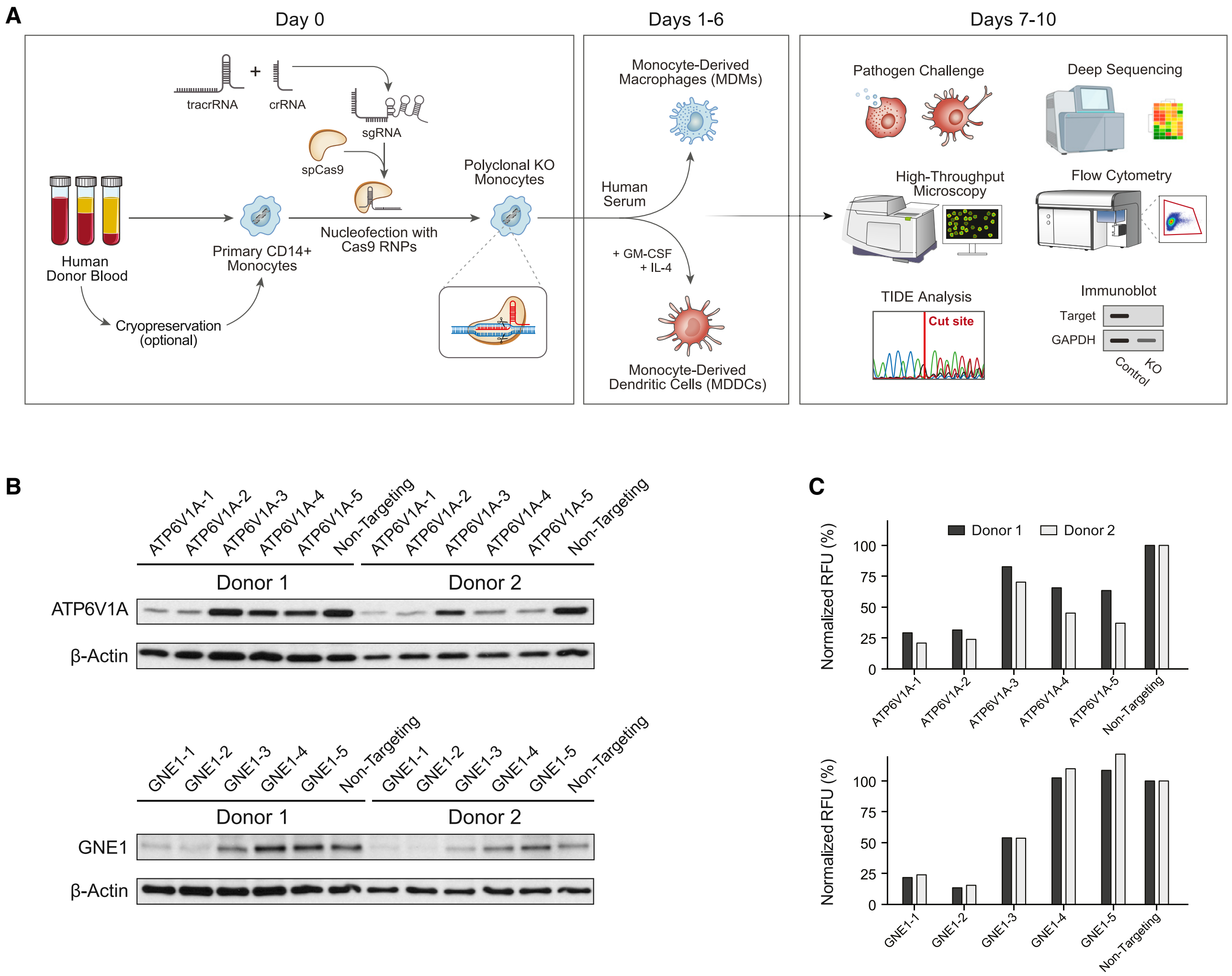

Figure 1. A flexible platform for CRISPR editing of human myeloid-lineage cells

(A) A generalized schematic of the platform. Human CD14$^+$ monocytes are isolated from blood by density gradient separation of PBMCs followed by magnetic negative selection. Either PBMCs or monocytes may be cryopreserved for later editing (Figure S1B). Cells are then nucleofected with preformed CRISPR-Cas9 RNPs and immediately put into differentiating culture under MDM- or MDDC-generating conditions. After allowing for 6–7 days of differentiation and washout of the targeted gene product, cells can be subjected to a wide variety of functional, phenotypic, and genotypic studies to assess the knockout efficiency and function of the targeted gene product.

(B) Guide sequence-dependent knockout of targeted genes leads to loss of gene products. CD14$^+$ monocytes were nucleofected with RNPs containing 1 of 5 distinct guide sequences against the indicated gene or a scrambled non-targeting control, cultured under MDM-generating conditions, and then lysed for immunoblot analysis. Blots show targeted gene protein product and untargeted housekeeping gene product β-actin protein levels in cells from 2 blood donors. GNE1 and ATP6V1A ran at their expected sizes of 79 and 69 kDa, respectively.

(C) Knockout was quantified by digital densitometry and normalized on a per-sample basis in relative fluorescence units (RFUs) to untargeted housekeeping control protein β-actin.

See also Figure S1.

both *CXCR4*-targeted and unperturbed MDDCs expressed high levels of *CD209* (*DC*-SIGN) and upregulated CD1 components and class II human leukocyte antigen gene complex (HLA) genes, consistent with differentiation into a professional antigen-presenting cell type. Although PMA-stimulated THP1 cells expressed some hallmark MDM transcripts, there remained a pronounced difference compared to any primary cell subset and they again segregated away from the differentiated primary cells even when clustered on only this set of transcripts, reinforcing the previous observation that differentiation is the primary driver of cell identity in these cells (Figure 2C). Hallmark gene set enrichment analysis (Liberzon et al., 2015; Subramanian et al., 2005) pointed to the decreased expression of protein secretion-related genes in Cas9 RNP nucleofected MDDCs as the largest and most significant change compared to unperturbed controls (Tables S1 and S2). Modest changes in Cas9 RNP nucleofected MDMs were linked to an increase in interferon response genes, notably *MX1* and *IFIT2* (Figure S2; Table S1). Overall, nucleofection did not

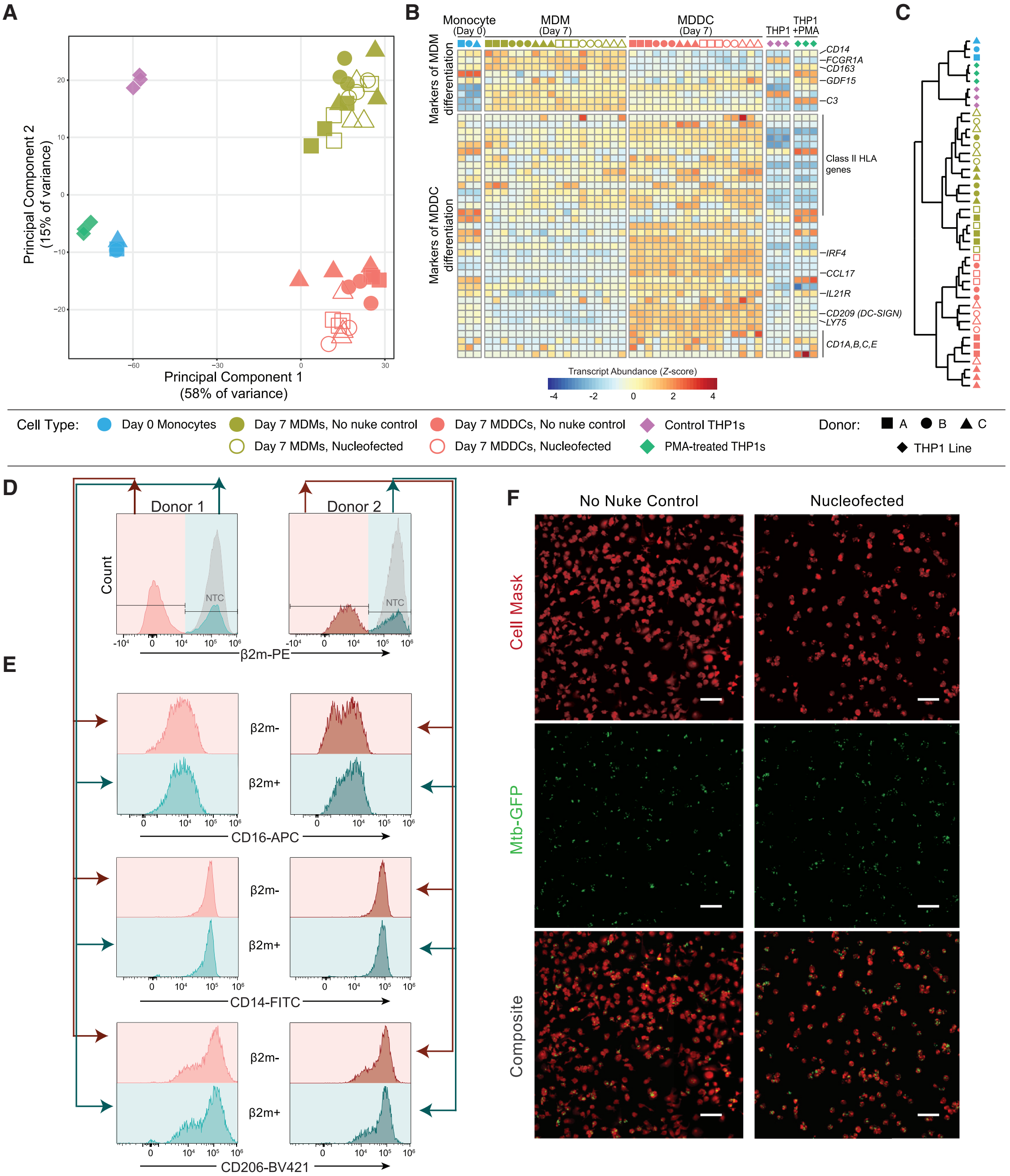

Figure 2. CRISPR-Cas9-mediated gene knockout preserves key aspects of differentiation and function in targeted myeloid cells

(A) Principal-component analysis of RNA sequencing (RNA-seq) from the indicated cell types.

(B) Normalized transcript abundance (*Z* score) for selected markers of MDM or MDDC differentiation (Lehtonen et al., 2007).

(C) Dendrogram of hierarchical clustering of the data in (B) by Euclidean distance.

(D and E) Among cells subjected to CRISPR-Cas9 RNP nucleofection, cell surface protein levels of CD16, CD14, and CD206 were compared between the cells that bear the desired β2 m knockout (pink) and those that do not (teal) by flow cytometry after 7 days of MDM differentiation. (D) shows gating, while (E) shows the expression of the indicated markers.

(F) Representative images of unperturbed (left) and RNP-nucleofected (right) MDMs infected with GFP-expressing *M. tuberculosis* (Mtb-GFP) show that CRISPR-Cas9-targeted cells remain competent to phagocytose living pathogens. Top, membrane staining with Cell Mask Far Red; CENTER, Mtb-GFP; bottom, composite. Scale bars represent 100 μm.

See also Figure S2.

prevent the induction of cell-type selective marker genes in MDMs and MDDCs.

We then asked whether editing selects for the survival of a subset of cells or whether editing alters differentiation in a nonspecific manner. We used an intermediate-efficiency guide against an easily stained surface antigen, β-2-microglobulin (β2m), to generate a roughly even mix of β2m^{-} and β2m^{+} cells that had been exposed to the same nucleofection and had been cultured together. These cells were subjected to MDM differentiation for 7 days before co-staining for β2m and several myeloid phenotypic markers (Figure 2D). The β2m^{+} cells thus served as in-well controls for the β2m^{-}, knockout cells. We observed no phenotypic difference between the β2m^{+} and β2m^{-} cells, suggesting that the process of CRISPR gene ablation did not appreciably select for a subset of cells, nor did it skew MDM differentiation.

Next, we assessed whether edited primary myeloid cells not only acquired key markers of differentiation but also retained critical functions of mature macrophages. We evaluated the ability of edited and unedited MDMs to phagocytose *Mycobacterium tuberculosis*, a deadly pathogen found inside macrophages and DCs during human infection (Wolf et al., 2007). Internalization of *M. tuberculosis*, a key stage in the bacterial life cycle, is mediated at least in part by phagocytosis by host macrophages (Ernst and Wolf, 2006; Srivastava et al., 2016). When we exposed either unperturbed MDMs or MDMs nucleofected with RNPs targeting the irrelevant *CXCR4* gene, we found that both cell populations were capable of phagocytosing the pathogens at a high rate, leaving few extracellular bacteria (Figure 2E). We again observed that nucleofection led to a loss of cells; when normalized for cell count, we found the rate of phagocytosis to be linear between nucleofected and unperturbed cells (Figures S3A–S3C). Thus, edited human myeloid cells can retain phagocytic ability, consistent with successful differentiation despite Cas9 RNP nucleofection, and represent a robust platform for functional assays.

Functional testing of host factors in isogenic primary myeloid cells

Finally, we sought to establish that this platform is suitable to test the function of genetic factors that control complex cellular behaviors. We targeted the host viral restriction factor SAMHD1, which blocks lentiviral infection (including HIV-1 infection) by depleting intracellular pools of deoxynucleoside triphosphates (dNTPs) needed for reverse transcription (St Gelais and Wu, 2011; Hrecka et al., 2011; Laguette et al., 2011; Sunseri et al., 2011). We used 5 distinct guides against *SAMHD1*, or a non-targeting control guide, to attempt to perturb the *SAMHD1* gene directly in primary human monocytes from 4 different blood donors. After 7 days of differentiation into MDMs, cells were exposed to the CCR5-tropic, chimeric HIV-1 clone LAI-YU2. Two days later, cells were stained with Hoechst dye and probed for the HIV-1 antigen p24, which is indicative of productive infection, then quantified by high-throughput microscopy (Figures 3A, 3B, S3D, and S3E). SAMHD1 deletion resulted in a significant, >50-fold increase in HIV infection. (guide SAMHD1-1: 51.74-fold increase in infection relative to non-targeting, 95% confidence interval [CI] 14.17–89.32; SAMHD1-2: 50.30-fold increase, 95% CI 12.73–87.88; SAMHD1-3: 39.17-fold increase, 95% CI 1.594–76.74; results of a repeated-measures 1-way ANOVA with Dunnett's multiple comparison test.) We observed a clear correlation between knockout efficiency as measured by protein or DNA (Figures 3C and 3D) and the degree of HIV infection, consistent with a causal relationship (Figure S3F). This dramatic effect upon ablation of a viral restriction factor clearly demonstrated that this platform is suitable for the functional assessment of host genes in primary human myeloid-lineage cells.

DISCUSSION

Building on previous work using CRISPR-Cas9 to render primary human immune cells genetically tractable (Hultquist et al., 2019; Hung et al., 2018; Nguyen et al., 2020; Schumann et al., 2015), we have developed a robust, flexible, and high-throughput-compatible platform for the genetic manipulation of primary human myeloid cells. We have optimized cell isolation, culture, and CRISPR-Cas9 RNP nucleofection conditions to allow for the rapid and inexpensive generation of isogenic modified cells that can then be differentiated and flexibly incorporated into downstream biochemical and phenotypic assays. Knockout can be easily achieved at programmed gene targets, and the resulting cells largely retain critical markers and key functional characteristics of differentiated myeloid cells. Differentiated, edited, monocyte-derived macrophages remain capable of phagocytosis of living pathogens, and we demonstrate that this editing system can be incorporated into existing assays to study complex biological phenotypes such as host-pathogen interactions.

This technology allows for the genetic interrogation of any type of primary cell that can be differentiated from a human blood monocyte. Although flexible, this also presents a limitation in types of myeloid cells; future work will be needed to make this protocol compatible with already-differentiated myeloid cell types such as alveolar or tissue-resident macrophages or circulating plasmacytoid DCs. More work will also be required to adapt this protocol for other Cas9-based tools and approaches (Hendriks et al., 2020; Nguyen et al., 2020; Roth et al., 2018; Shifrut et al., 2018); while we only demonstrate knockout, this efficient nucleofection of Cas9 RNPs into cells is likely to be a fruitful foundation for other genome or epigenome modification strategies.

The platform we present here improves significantly upon the existing technology to manipulate human myeloid cells. Genetic perturbation to date has rested largely upon RNAi technologies, which compared to CRISPR-based approaches can have higher off-target effects and result in transient, incomplete knockdown, rather than knockout (Housden and Perrimon, 2016), or upon lentiviral transduction, which is inefficient in these cells due to high expression of SAMHD1 (St Gelais and Wu, 2011; Goujon et al., 2007). This has hampered both the mechanistic investigation of these important, functionally diverse cells and the development of strategies to use these cells as therapeutics. For these reasons, other groups have previously sought workarounds to make myeloid cells genetically tractable. One approach is to edit hematopoietic progenitor/stem cells and differentiate them into myeloid lineage cells (Kang et al., 2015; Lee et al., 2018; Sontag et al., 2017). Comparatively, this route is difficult, expensive, and time-consuming (Sugimura et al., 2017). Another approach is to edit fully differentiated macrophages, which has demonstrated some functional success in the literature (Barkal et al., 2018; Haney et al., 2018), although this also has limitations. The approach is

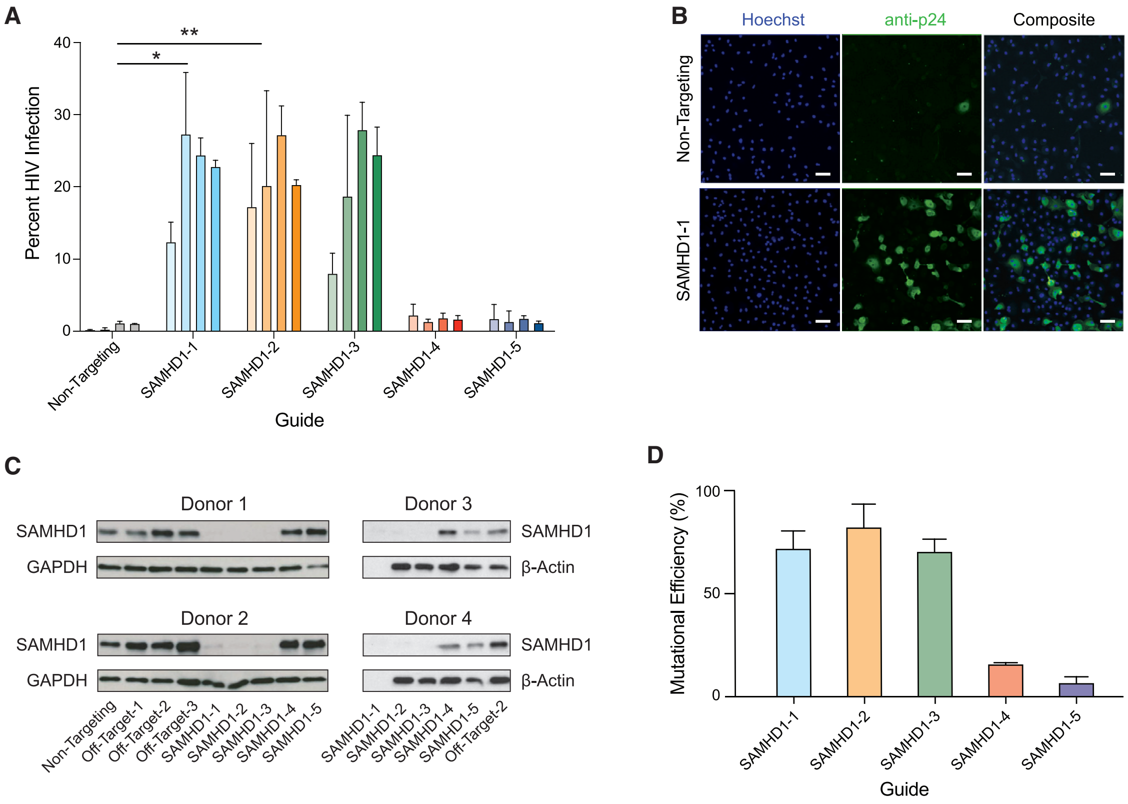

Figure 3. Generation of isogenic monocyte-derived macrophages for functional evaluation of an HIV-1 host restriction factor

(A) *SAMHD1*-targeted or non-targeting control MDMs from 4 independent, HIV$^-$ blood donors were infected with HIV-1. The plot displays the percentage of cells productively infected after a 48-h exposure. Guides that most efficiently ablated the gene caused statistically significant increases in infection as assessed by 1-way ANOVA followed by Dunnett's test. *$p < 0.05$, **$p < 0.01$. See also Figure S3E.

(B) Representative images of HIV-1 infection from donor 3 comparing cells nucleofected with control non-targeting RNPs (top) to cells nucleofected with RNPs made from guide SAMHD1-1 (bottom). Left, Hoechst; center, staining of the HIV-1 antigen p24; right, composite. Scale bars represent 100 μm. For representative images of all of the guides, see Figure S3D.

(C and D) Quantification of *SAMHD1* knockout by immunoblot (C) and sequencing (D). No protein sample was available for guide SAMHD1-1 in donors 3 and 4; Sanger sequencing was analyzed for mutational efficiency by TIDE, bars represent means ± SDs for at least 3 biological replicates.

See also Figure S3.

specific to macrophages, rather than allowing for flexible differentiation, and is limited by the lifespan of mature macrophages in culture. As a result, phenotypic assessment has been limited to short timescale assays soon after editing, when cells display appropriate levels of genetic editing but may still have variable concentrations of the protein product of a targeted gene remaining in the cell.

This platform for editing primary human monocytes before differentiation ameliorates these previous limitations. Cells may be differentiated into MDDCs or MDMs; knockout is robust, permanent, and can be rapidly and inexpensively iterated by substituting guide RNA sequences; there is adequate time for turnover of the targeted gene product and for subsequent multi-day functional and phenotypic assessment; and the cells are generated in only 1 week. Isolated cells can be cryopreserved before editing, allowing additional experimental flexibility. Furthermore, all of the reagents and equipment are readily available. We anticipate that these features, which have proven key to the success of CRISPR-Cas9 RNP-based editing of T cells, will facilitate a diverse set of research endeavors and hopefully accelerate the development of myeloid cell therapies.

STAR★METHODS

Detailed methods are provided in the online version of this paper and include the following:

- KEY RESOURCES TABLE
- RESOURCE AVAILABILITY
  - Lead contact
  - Materials availability
  - Data and code availability
- EXPERIMENTAL MODEL AND SUBJECT DETAILS
- METHOD DETAILS
  - Primary human monocyte isolation and enrichment
  - Formation of Cas9-ribonucleoproteins
  - Nucleofection of Cas9-ribonucleoproteins into primary human monocytes

- ○ *In vitro* differentiation of monocyte-derived macrophages and dendritic cells
- ○ Flow cytometric staining and analysis
- ○ *In vitro* infection of MDMs by *Mycobacterium tuberculosis*
- ○ Quantification of *Mycobacterium tuberculosis* infection
- ○ HIV production
- ○ HIV infection, p24 staining and imaging
- ○ Immunoblotting and protein quantification
- ○ Quantification of mutational efficiency by TIDE analysis
- ○ Analysis of cell survival by luminescence
- ○ RNA-seq analysis
- QUANTIFICATION AND STATISTICAL ANALYSIS

SUPPLEMENTAL INFORMATION

Supplemental information can be found online at https://doi.org/10.1016/j.celrep.2021.109105.

ACKNOWLEDGMENTS

The authors acknowledge their partners and families for supporting us during this work. We thank all of the members of the Marson lab and the Krogan lab for helpful conversations and advice. We are grateful for discussions with B.R. Conklin, M. Ott, D.G. Russel, M. Jost, V. Ramani, G. Alberts, S. Pyle, D. Sainz, and G. Ehle. We are grateful for the generosity of our blood donors and the help of Y. Dayter and the research support team at Vitalant. R. Gummuluru and S. Stanley graciously shared the HIV-1 plasmid and the Mtb-GFP bacteria, respectively. Parts of Figure 1A were created with BioRender.com. The RNA sequencing was carried out by V. Rashbrook, S. Ashtari, and C. Goldman at the DNA Technologies and Expression Analysis Core at the University of California, Davis (UCSD) Genome Center, supported by NIH Shared Instrumentation Grant 1S10OD010786-01. J.H. was supported by the UCSF MSTP (T32GM007618). K.M.H. is supported by the National Science Foundation (1650113). Y.L. was supported by the Ruth L. Kirschstein Fellowship (T32AI00733429). The Marson lab has received gifts from J. Aronov, G. Hoskin, K. Jordan, B. Bakar, and the Caufield family, and has received funds from the Gladstone Institutes, the Innovative Genomics Institute (IGI), and the Parker Institute for Cancer Immunotherapy (PICI). A.M. holds a Career Award for Medical Scientists from the Burroughs Wellcome Fund, the Lloyd J. Old STAR award from the Cancer Research Institute (CRI), and is an investigator at the Chan Zuckerberg Biohub. The Krogan Laboratory has received research support from Vir Biotechnology and F. Hoffmann-La Roche. The Marson, Krogan, and Cox labs have received funding from the BioFulcrum Viral and Infectious Disease Research Program. This work was supported by funding from the James B. Pendleton Charitable Trust and by NIH grants P50 AI150476 (A.M. and N.J.K.), U19 AI135990 (A.M., N.J.K., and J.S.C.), P01 AI063302 (N.J.K. and J.S.C), R01 AI150449 (S.K.P.), and R01 AI124471 (J.D.E).

AUTHOR CONTRIBUTIONS

Conceptualization, J.H., D.A.C., M.J.M., T.L.R., M.S.B., E.V.D., K.A.F., S.K.P., N.J.K., and A.M. Investigation, J.H., D.A.C., M.J.M., V.E.K., D.E.G., W.Z., J.M.B., K.M.H., U.R., A.M.-F., and J.A.W. Resources, A.M.-F., K.A.F., J.S.C., J.D.E., N.J.K., and A.M. Formal analysis, J.H., D.A.C., M.J.M., D.W., and E.S. Supervision, J.H., K.A.F., J.S.C., J.D.E., N.J.K., and A.M. Funding acquisition, K.A.F., J.S.C., J.D.E., N.J.K., and A.M. Writing – original draft, J.H., D.A.C., M.J.M., and A.M. Writing – review & editing, J.H., D.A.C., M.J.M., W.Z., T.L.R., K.M.H., Y.L., M.S.B., J.F.H., J.A.W., K.A.F., J.D.E., N.J.K., and A.M.

DECLARATION OF INTERESTS

The authors declare competing interests: T.L.R. is a co-founder of Arsenal Biosciences. A.M. is a compensated co-founder, member of the boards of directors, and a member of the scientific advisory boards of Spotlight Therapeutics and Arsenal Biosciences. A.M. was a compensated member of the scientific advisory board at PACT Pharma and was a compensated advisor to Juno Therapeutics and Trizell. A.M. owns stock in Arsenal Biosciences, Spotlight Therapeutics, and PACT Pharma. A.M. has received honoraria from Merck and Vertex, a consulting fee from AlphaSights, and is an investor in and informal advisor to Offline Ventures. The Marson lab has received research support from Juno Therapeutics, Epinomics, Sanofi, GlaxoSmithKline, Gilead, and Anthem. A.M., T.L.R., and E.S. are holders of patents pertaining to, but not resulting from, this work. The Krogan laboratory has received research support from Vir Biotechnology and F. Hoffmann-La Roche.

Received: March 10, 2020
Revised: December 31, 2020
Accepted: April 19, 2021
Published: May 11, 2021

REFERENCES

Auffray, C., Sieweke, M.H., and Geissmann, F. (2009). Blood monocytes: development, heterogeneity, and relationship with dendritic cells. Annu. Rev. Immunol. *27*, 669–692.

Barkal, A.A., Weiskopf, K., Kao, K.S., Gordon, S.R., Rosental, B., Yiu, Y.Y., George, B.M., Markovic, M., Ring, N.G., Tsai, J.M., et al. (2018). Engagement of MHC class I by the inhibitory receptor LILRB1 suppresses macrophages and is a target of cancer immunotherapy. Nat. Immunol. *19*, 76–84.

Brinkman, E.K., Chen, T., Amendola, M., and van Steensel, B. (2014). Easy quantitative assessment of genome editing by sequence trace decomposition. Nucleic Acids Res. *42*, e168.

Chao, M.P., Takimoto, C.H., Feng, D.D., McKenna, K., Gip, P., Liu, J., Volkmer, J.-P., Weissman, I.L., and Majeti, R. (2020). Therapeutic Targeting of the Macrophage Immune Checkpoint CD47 in Myeloid Malignancies. Front. Oncol. *9*, 1380.

Engblom, C., Pfirschke, C., and Pittet, M.J. (2016). The role of myeloid cells in cancer therapies. Nat. Rev. Cancer *16*, 447–462.

Ernst, J.D., and Wolf, A. (2006). Phagocytosis of Bacteria and Bacterial Pathogenicity (Cambridge University Press), pp. 246–272.

Figueroa, G., Parira, T., Laverde, A., Casteleiro, G., El-Mabhouh, A., Nair, M., and Agudelo, M. (2016). Characterization of Human Monocyte-derived Dendritic Cells by Imaging Flow Cytometry: A Comparison between Two Monocyte Isolation Protocols. J. Vis. Exp. (*116*), 54296.

Foss, D.V., Hochstrasser, M.L., and Wilson, R.C. (2019). Clinical applications of CRISPR-based genome editing and diagnostics. Transfusion *59*, 1389–1399.

Geissmann, F., and Mass, E. (2015). A stratified myeloid system, the challenge of understanding macrophage diversity. Semin. Immunol. *27*, 353–356.

Germic, N., Frangez, Z., Yousefi, S., and Simon, H.-U. (2019). Regulation of the innate immune system by autophagy: monocytes, macrophages, dendritic cells and antigen presentation. Cell Death Differ. *26*, 715–727.

Gordon, D.E., Hiatt, J., Bouhaddou, M., Rezelj, V.V., Ulferts, S., Braberg, H., Jureka, A.S., Obernier, K., Guo, J.Z., Batra, J., et al.; QCRG Structural Biology Consortium; Zoonomia Consortium (2020). Comparative host-coronavirus protein interaction networks reveal pan-viral disease mechanisms. Science *370*, eabe9403.

Goujon, C., Rivière, L., Jarrosson-Wuilleme, L., Bernaud, J., Rigal, D., Darlix, J.-L., and Cimarelli, A. (2007). SIVSM/HIV-2 Vpx proteins promote retroviral escape from a proteasome-dependent restriction pathway present in human dendritic cells. Retrovirology *4*, 2.

Han, J., Perez, J.T., Chen, C., Li, Y., Benitez, A., Kandasamy, M., Lee, Y., Andrade, J., tenOever, B., and Manicassamy, B. (2018). Genome-wide CRISPR/Cas9 Screen Identifies Host Factors Essential for Influenza Virus Replication. Cell Rep. *23*, 596–607.

Hancock, D.G., Guy, T.V., Shklovskaya, E., and Fazekas de St Groth, B. (2013). Experimental models to investigate the function of dendritic cell subsets: challenges and implications. Clin. Exp. Immunol. *171*, 147–154.

Haney, M.S., Bohlen, C.J., Morgens, D.W., Ousey, J.A., Barkal, A.A., Tsui, C.K., Ego, B.K., Levin, R., Kamber, R.A., Collins, H., et al. (2018). Identification of phagocytosis regulators using magnetic genome-wide CRISPR screens. Nat. Genet. *50*, 1716–1727.

Hendriks, D., Clevers, H., and Artegiani, B. (2020). CRISPR-Cas Tools and Their Application in Genetic Engineering of Human Stem Cells and Organoids. Cell Stem Cell *27*, 705–731.

Housden, B.E., and Perrimon, N. (2016). Comparing CRISPR and RNAi-based screening technologies. Nat. Biotechnol. *34*, 621–623.

Hrecka, K., Hao, C., Gierszewska, M., Swanson, S.K., Kesik-Brodacka, M., Srivastava, S., Florens, L., Washburn, M.P., and Skowronski, J. (2011). Vpx relieves inhibition of HIV-1 infection of macrophages mediated by the SAMHD1 protein. Nature *474*, 658–661.

Hultquist, J.F., Hiatt, J., Schumann, K., McGregor, M.J., Roth, T.L., Haas, P., Doudna, J.A., Marson, A., and Krogan, N.J. (2019). CRISPR-Cas9 genome engineering of primary $CD4^+$ T cells for the interrogation of HIV-host factor interactions. Nat. Protoc. *14*, 1–27.

Hung, K.L., Meitlis, I., Hale, M., Chen, C.-Y., Singh, S., Jackson, S.W., Miao, C.H., Khan, I.F., Rawlings, D.J., and James, R.G. (2018). Engineering Protein-Secreting Plasma Cells by Homology-Directed Repair in Primary Human B Cells. Mol. Ther. *26*, 456–467.

Jin, X., and Kruth, H.S. (2016). Culture of Macrophage Colony-stimulating Factor Differentiated Human Monocyte-derived Macrophages. J. Vis. Exp. (*112*), 54244.

Kang, H., Minder, P., Park, M.A., Mesquitta, W.-T., Torbett, B.E., and Slukvin, I.I. (2015). CCR5 Disruption in Induced Pluripotent Stem Cells Using CRISPR/Cas9 Provides Selective Resistance of Immune Cells to CCR5-tropic HIV-1 Virus. Mol. Ther. Nucleic Acids *4*, e268.

Laguette, N., Sobhian, B., Casartelli, N., Ringeard, M., Chable-Bessia, C., Ségéral, E., Yatim, A., Emiliani, S., Schwartz, O., and Benkirane, M. (2011). SAMHD1 is the dendritic- and myeloid-cell-specific HIV-1 restriction factor counteracted by Vpx. Nature *474*, 654–657.

Lapenna, A., De Palma, M., and Lewis, C.E. (2018). Perivascular macrophages in health and disease. Nat. Rev. Immunol. *18*, 689–702.

Lee, C.Z.W., Kozaki, T., and Ginhoux, F. (2018). Studying tissue macrophages in vitro: are iPSC-derived cells the answer? Nat. Rev. Immunol. *18*, 716–725.

Lehtonen, A., Ahlfors, H., Veckman, V., Miettinen, M., Lahesmaa, R., and Julkunen, I. (2007). Gene expression profiling during differentiation of human monocytes to macrophages or dendritic cells. J. Leukoc. Biol. *82*, 710–720.

Liberzon, A., Birger, C., Thorvaldsdóttir, H., Ghandi, M., Mesirov, J.P., and Tamayo, P. (2015). The Molecular Signatures Database (MSigDB) hallmark gene set collection. Cell Syst. *1*, 417–425.

Medzhitov, R., and Janeway, C.A., Jr. (1997). Innate immunity: the virtues of a nonclonal system of recognition. Cell *91*, 295–298.

Medzhitov, R., and Janeway, C., Jr. (2000). Innate immunity. N. Engl. J. Med. *343*, 338–344.

Nguyen, D.N., Roth, T.L., Li, P.J., Chen, P.A., Apathy, R., Mamedov, M.R., Vo, L.T., Tobin, V.R., Goodman, D., Shifrut, E., et al. (2020). Polymer-stabilized Cas9 nanoparticles and modified repair templates increase genome editing efficiency. Nat. Biotechnol. *38*, 44–49.

Norton, T.D., Miller, E.A., Bhardwaj, N., and Landau, N.R. (2015). Vpx-containing dendritic cell vaccine induces CTLs and reactivates latent HIV-1 in vitro. Gene Ther. *22*, 227–236.

Qin, Z. (2012). The use of THP-1 cells as a model for mimicking the function and regulation of monocytes and macrophages in the vasculature. Atherosclerosis *221*, 2–11.

R Development Core Team (2020). R: a language and environment for statistical computing (R Foundation for Statistical Computing).

Roth, T.L., Puig-Saus, C., Yu, R., Shifrut, E., Carnevale, J., Li, P.J., Hiatt, J., Saco, J., Krystofinski, P., Li, H., et al. (2018). Reprogramming human T cell function and specificity with non-viral genome targeting. Nature *559*, 405–409.

Schindelin, J., Arganda-Carreras, I., Frise, E., Kaynig, V., Longair, M., Pietzsch, T., Preibisch, S., Rueden, C., Saalfeld, S., Schmid, B., et al. (2012). Fiji: an open-source platform for biological-image analysis. Nat. Methods *9*, 676–682.

Schumann, K., Lin, S., Boyer, E., Simeonov, D.R., Subramaniam, M., Gate, R.E., Haliburton, G.E., Ye, C.J., Bluestone, J.A., Doudna, J.A., and Marson, A. (2015). Generation of knock-in primary human T cells using Cas9 ribonucleoproteins. Proc. Natl. Acad. Sci. USA *112*, 10437–10442.

Shifrut, E., Carnevale, J., Tobin, V., Roth, T.L., Woo, J.M., Bui, C.T., Li, P.J., Diolaiti, M.E., Ashworth, A., and Marson, A. (2018). Genome-wide CRISPR Screens in Primary Human T Cells Reveal Key Regulators of Immune Function. Cell *175*, 1958–1971.e15.

Simeonov, D.R., and Marson, A. (2019). CRISPR-Based Tools in Immunity. Annu. Rev. Immunol. *37*, 571–597.

Sontag, S., Förster, M., Qin, J., Wanek, P., Mitzka, S., Schüler, H.M., Koschmieder, S., Rose-John, S., Seré, K., and Zenke, M. (2017). Modelling IRF8 Deficient Human Hematopoiesis and Dendritic Cell Development with Engineered iPS Cells. Stem Cells *35*, 898–908.

Srivastava, S., Grace, P.S., and Ernst, J.D. (2016). Antigen Export Reduces Antigen Presentation and Limits T Cell Control of M. tuberculosis. Cell Host Microbe *19*, 44–54.

St Gelais, C., and Wu, L. (2011). SAMHD1: a new insight into HIV-1 restriction in myeloid cells. Retrovirology *8*, 55.

Stadtmauer, E.A., Fraietta, J.A., Davis, M.M., Cohen, A.D., Weber, K.L., Lancaster, E., Mangan, P.A., Kulikovskaya, I., Gupta, M., Chen, F., et al. (2020). CRISPR-engineered T cells in patients with refractory cancer. Science *367*, eaba7365.

Subramanian, A., Tamayo, P., Mootha, V.K., Mukherjee, S., Ebert, B.L., Gillette, M.A., Paulovich, A., Pomeroy, S.L., Golub, T.R., Lander, E.S., and Mesirov, J.P. (2005). Gene set enrichment analysis: a knowledge-based approach for interpreting genome-wide expression profiles. Proc. Natl. Acad. Sci. USA *102*, 15545–15550.

Sugimura, R., Jha, D.K., Han, A., Soria-Valles, C., da Rocha, E.L., Lu, Y.-F., Goettel, J.A., Serrao, E., Rowe, R.G., Malleshaiah, M., et al. (2017). Haematopoietic stem and progenitor cells from human pluripotent stem cells. Nature *545*, 432–438.

Sunseri, N., O'Brien, M., Bhardwaj, N., and Landau, N.R. (2011). Human immunodeficiency virus type 1 modified to package Simian immunodeficiency virus Vpx efficiently infects macrophages and dendritic cells. J. Virol. *85*, 6263–6274.

Tsuchiya, S., Yamabe, M., Yamaguchi, Y., Kobayashi, Y., Konno, T., and Tada, K. (1980). Establishment and characterization of a human acute monocytic leukemia cell line (THP-1). Int. J. Cancer *26*, 171–176.

Tsuchiya, S., Kobayashi, Y., Goto, Y., Okumura, H., Nakae, S., Konno, T., and Tada, K. (1982). Induction of maturation in cultured human monocytic leukemia cells by a phorbol diester. Cancer Res. *42*, 1530–1536.

Watson, R.O., Bell, S.L., MacDuff, D.A., Kimmey, J.M., Diner, E.J., Olivas, J., Vance, R.E., Stallings, C.L., Virgin, H.W., and Cox, J.S. (2015). The Cytosolic Sensor cGAS Detects Mycobacterium tuberculosis DNA to Induce Type I Interferons and Activate Autophagy. Cell Host Microbe *17*, 811–819.

Wolf, A.J., Linas, B., Trevejo-Nuñez, G.J., Kincaid, E., Tamura, T., Takatsu, K., and Ernst, J.D. (2007). Mycobacterium tuberculosis infects dendritic cells with high frequency and impairs their function in vivo. J. Immunol. *179*, 2509–2519.

Worbs, T., Hammerschmidt, S.I., and Förster, R. (2017). Dendritic cell migration in health and disease. Nat. Rev. Immunol. *17*, 30–48.

Wu, Y., Zeng, J., Roscoe, B.P., Liu, P., Yao, Q., Lazzarotto, C.R., Clement, K., Cole, M.A., Luk, K., Baricordi, C., et al. (2019). Highly efficient therapeutic gene editing of human hematopoietic stem cells. Nat. Med. *25*, 776–783.

Wynn, T.A., Chawla, A., and Pollard, J.W. (2013). Macrophage biology in development, homeostasis and disease. Nature *496*, 445–455.

CellPress
OPEN ACCESS

STAR★METHODS

KEY RESOURCES TABLE

REAGENT or RESOURCE	SOURCE	IDENTIFIER
Antibodies		
Mouse anti-human CD206 BV421 (clone 19.2)	Becton Dickinson	Cat# 566281; RRID:AB_2739655
Mouse anti-human CD11c PerCP-Cy5.5 (clone B-ly6)	Becton Dickinson	Cat# 565227; RRID:AB_2739122
Mouse anti-human CD16 APC (clone 3G8)	Biolegend	Cat# 302012; RRID:AB_314212
Mouse anti-human CD11b BV650 (clone ICRF44)	Becton Dickinson	Cat# 740566; RRID:AB_2740267
Mouse anti-human CD14 FITC (clone M5E2)	Biolegend	Cat# 301804; RRID:AB_314186
Mouse anti-human β2-Microglobulin PE (clone TU99)	Becton Dickinson	Cat# 551337; RRID:AB_394152
Mouse anti-human HLA-DR Pacific Blue (TU36)	Life Technologies	Cat# MHLDR28; RRID: AB_10401403
Human anti-human CD14 PE (clone REA599)	Miltenyi Biotec	Cat# 130-110-519; RRID:AB_2655051
Human anti-human CD16 APC (clone REA423)	Miltenyi Biotech	Cat# 130-106-705; RRID:AB_2655406
Ghost Dye™ Violet 510	Tonbo Biosciences	Cat# 13-0870-T100
Ghost Dye™ Violet 780	Tonbo Biosciences	Cat# 13-0865-T100
Human TruStain FcX (Fc Receptor Blocking Solution)	Biolegend	Cat# 422302
Rabbit recombinant monoclonal Anti-ATP6V1A	Abcam	Cat# EPR19270; RRID: AB_199326
Rabbit polyclonal anti-CSE1L	Proteintech	Cat# 22219-1-AP; RRID:AB_10950892
Rabbit polyclonal anti-GNE	Proteintech	Cat# 25079-1-AP
Rabbit monoclonal anti-β-Actin	Cell Signaling Technology	Cat# 4970P; RRID:AB_2223172
Mouse monoclonal anti-GAPDH	Sigma	Cat# G8795; RRID:AB_1078991
Mouse monoclonal anti-p24	AIDS Reagent	Cat# 183-H12-5C; RRID:AB_2819250
Rabbit polyclonal anti-SAMHD1	Proteintech	Cat# 12586-1-AP; RRID:AB_2183496
Donkey anti-mouse Alexa Fluor 488	Invitrogen	Cat# A-21202; RRID:AB_141607
Bacterial and virus strains		
HIV LAI-YU2	Gummuluru, Boston University	N/A
M. tuberculosis (Erdman Strain pMV261-eGFP)	Stanley Lab, UC Berkeley	ATCC #35801
M. bovis bacillus Calmette-Guerin (BCG) δAg85B/Ag85B-mCherry	Ernst Lab, UCSF	N/A
Biological samples		
Leukoreduction chambers from healthy human donors	Vitalant	N/A
1/10 Leukapheresis pack	STEMCELL	200-0092
Chemicals, peptides, and recombinant proteins		
Cas9-NLS purified protein	QB3 Macrolab	N/A
GM-CSF Human Recombinant	Life Technologies	Cat# PHC2015

(*Continued on next page*)

Continued		
REAGENT or RESOURCE	SOURCE	IDENTIFIER
IL-4 Human Recombinant Protein	Life Technologies	Cat# PHC0045
Triton™ X-100 (for molecular biology)	Sigma-Aldrich	Cat# T8787
NaCl	Corning	Cat# 46-032-CV
PEG-6000	Millipore Sigma	Cat# 8074911000
Glycerol	Sigma-Aldrich	Cat# G7893
Tween-80	N/A	N/A
PolyJet	Signagen	Cat# SL100688
Penicillin/Streptomycin	Corning	Cat# 30-002-CI
Ficoll-Paque Density Gradient	Sigma-Aldrich	Cat# GE17-1440-03
Hygromycin B	N/A	N/A
Kanamycin	N/A	N/A
Albumin	Sigma	Cat# A1470-100G
Dextrose	Fisher	Cat# D16500
Catalase	Sigma	Cat# C9322-5G
Saponin	Sigma	Cat# 47036
Phusion Hot Start 2X Master Mix	New England Biolabs	Cat# M0536L
HCS CellMask™ Green Stain	ThermoFisher Scientific	Cat# H32714
HCS CellMask™ Deep Red Stain	ThermoFisher Scientific	Cat# H32721
Hoechst 33258	Sigma-Aldrich	Cat# 861405
RPMI 1640 with 1x L-Glutamine	Corning	MT10040CV
HEPES	VWR	16777-032
Sodium Pyruvate	Fisher	MT 25-000-CI
PMA	Fisher	BP685-1
Critical commercial assays		
StemCell™ Human Monocyte Isolation Kit	STEMCELL Technologies	Cat# 19359
P2 Primary Cell 4D-Nucleofector™ X Kit L	Lonza	Cat# V4XP-2024
CellTiter-Glo Luminescent Cell Viability Assay	Promega	Cat# G7570
RNeasy 96 QIAcube HT Kit	QIAGEN	Cat# 74171
QuantSeq 3′ mRNA-seq Library Prep Kit FWD for Illumina	Lexogen	Cat# 015
Experimental models: cell lines		
Homo sapiens: 293T/17	UCSF CCF	N/A
Homo sapiens: THP-1	ATCC	TIB-202
Oligonucleotides		
Primer: PPIA Forward TGTTGACAGGGTGGTGACTTCA	IDT	N/A
Primer: PPIA Reverse ACTTAATTggttgggcgcagtg	IDT	N/A
Primer: CXCR4 Forward AGAGGAGTTAGCCAAGATGTGAC TTTGAAACC	IDT	N/A
Primer: CXCR4 Reverse GGACAGGATGACAATACCAGGCAG GATAAGGCC	IDT	N/A
Primer: CCR5 Forward TGCTTGGCCAAAAAGAGAGTTA	IDT	N/A
Primer: CCR5 Reverse TTTAAAGCAAACACAGCATGGA	ID T	N/A

(Continued on next page)

Continued

REAGENT or RESOURCE	SOURCE	IDENTIFIER
Primer: SAMHD1-01 Forward #1 GTAGCCATGCAGCGAGCCGATT	I DT	N/A
Primer: SAMHD1-01 Reverse #1 AGGGACCCGAGTCTCGCTTGTC	IDT	N/A
Primer: SAMHD1-01 Forward #2 TTTGAGGACGACTGGACTGC	IDT	N/A
Primer: SAMHD1-01 Reverse #2 CTCCCATCCTACGAATCGCC	IDT	N/A
Primer: SAMHD1-02 Forward CGGTGGAGAAGCAGTTGTCT	IDT	N/A
Primer: SAMHD1-02 Reverse TGGGAAGCTAAAATCGTTCCA	IDT	N/A
Primer: SAMHD1-03 Forward TCAAATAGCTTTGACTTTGCAC	IDT	N/A
Primer: SAMHD1-03 Reverse GCCTCAATTTTCTCATCAATAAA	IDT	N/A
Primer: SAMHD1-04 Forward ACATCTTGTCATTTCCGTTAGT	IDT	N/A
Primer: SAMHD1-04 Reverse GCCTCAATTTTCTCATCAATAAAA	IDT	N/A
Primer: SAMHD1-05 Forward TGGCTTTACTAATCTGCCTCCTCA	IDT	N/A
Primer: SAMHD1-05 Reverse TCACGGAGAGACCTGGCTGT	IDT	N/A
tracrRNA	Dharmacon	Cat# U-002005-0050
Non-targeting crRNA GTCGACGTTATTGCCGGTCG	Dharmacon	Cat# U-007503-01-0020
SAMHD1 crRNA 1 GTGCTGCTGAAGAACATCCG	Dharmacon	Cat# CM-013950-01-0020
SAMHD1 crRNA 2 CTTACCTGTCAGCTTAGTAT	Dharmacon	Cat# CM-013950-02-0020
SAMHD1 crRNA 3 CGATACATCAAACAGCTGGG	Dharmacon	Cat# CM-013950-03-0020
SAMHD1 crRNA 4 GTGTATCAATGATTCGGACG	Dharmacon	Cat# CM-013950-04-0020
SAMHD1 crRNA 5 GGTGTAAAGAGTTGCGAGTG	Dharmacon	Cat# CM-013950-05-0020
CXCR4 crRNA GAAGCGTGATGACAAAGAGG	Dharmacon	N/A (Custom synthesis)
CCR5 crRNA CCTGCCTCCGCTCTACTCAC	Dharmacon	N/A (Custom synthesis)
β2-microglobulin crRNA GAGTAGCGCGAGCACAGCTA	Dharmacon	N/A (Custom synthesis)
PPIA crRNA ACTGCCAAGACTGAGTGGTA	Dharmacon	CM-004979-03-0020
AAVS1 crRNA GTCACCAATCCTGTCCCTAG	Dharmacon	N/A (Custom synthesis)
Recombinant DNA		
HIV LAI-YU2 plasmid	Gummuluru Lab, Boston University	N/A
Software and algorithms		
GraphPad Prism v8.3.0	GraphPad Software, LLC	RRID: SCR_002798 https://www.graphpad.com
TIDE: Tracking of Indels by DEcomposition	(Brinkman et al., 2014)	http://tide.nki.nl/

(*Continued on next page*)

Continued

REAGENT or RESOURCE	SOURCE	IDENTIFIER
Benchling	Benchling [Molecular Biology]. (2020).	https://www.benchling.com
BioRender	BioRender. (2020).	https://www.biorender.com
ImageJ	https://imagej.nih.gov/ij/	RRID: SCR_003070 https://imagej.nih.gov/ij/
FlowJo 10 v10.6.1	Becton Dickinson	RRID: SCR_008520 https://www.flowjo.com
Primer3		https://primer3.org
Attune™ NxT Software v3.1.2	Life Technologies	https://www.thermofisher.com/us/en/home/life-science/cell-analysis/flow-cytometry/flow-cytometers/attune-acoustic-focusing-flow-cytometer/attune-cytometer-software.html
HCS Studio™ Cell Analysis Software v6.6.0	ThermoFisher Scientific	Cat# SX00041A
R: A Language and Environment for Statistical Computing	R Development Core Team (2020)	www.R-project.org/
Other		
Iscove's Modified Dulbecoo's Medium	GIBCO	Cat# 12440053
RPMI 1640 Medium	GIBCO	Cat# 11-875-093
Heat Inactivated Human AB Serum (lot 7J2616)	Valley Biomedical Products & Services	Cat# HP1022HI
Fetal Bovine Serum, Qualified One Shot™	GIBCO	Cat# A3160502
4–20% Criterion TGX Gel	Bio-Rad	Cat# 567-1094
PVDF Membrane	Bio-Rad	Cat# 1620177
HyBlot CL® Audiogradiography Film	Thomas Scientific	Cat# 1159T41
Pierce™ ECL Western Blotting Substrate	ThermoFisher Scientific	Cat# 32106
7H9 Middlebrook Medium	BD Difco	Cat# 271310
Middlebrook OADC Growth Supplement	Sigma-Aldrich	Cat# M0678
UltraComp eBeads Compensation Beads	Life Technologies	Cat# 01-2222-42
Accutase Enzyme Cell Detachment Medium	ThermoFisher Scientific (Invitrogen™)	Cat# 00-4555-56
Falcon® 96-well Clear Flat Bottom Not Treated Cell Culture Microplate, With Lid, Sterile	Corning	Cat# 351178
Falcon® 96-well Clear Flat Bottom Not Treated Cell Culture Microplate, With Lid, Sterile	Corning	Cat# 351172
TempPlate Non-Skirted 96-well PCR Plate	USA Scientific	Cat# 1402-9598
Konica Minolta SRX-101A Medical Film Processor	Konica Minolta	Cat# SRX-101A

RESOURCE AVAILABILITY

Lead contact

Questions and requests for resources and reagents should be directed to the Lead Contact, Dr. Alexander Marson (Alexander.Marson@ucsf.edu).

Materials availability

All materials used in this study are commercially available, please see the Key Resources Table. No unique reagents were generated in this study.

Data and code availability

Code was generated only for formatting and visualization of data; all code is available upon reasonable request. Sequencing files are available via SRA as BioProject PRJNA684421.

EXPERIMENTAL MODEL AND SUBJECT DETAILS

Human 293T/17 cells were obtained from the UCSF Cell Culture Facility and were cultured in Dulbecco's Modified Eagle Medium (DMEM) supplemented with 10% fetal calf serum (FCS, Invitrogen 26140079) at 37°C in 5% CO_2. Human peripheral blood mononuclear cells (PBMC) were isolated from TRIMA residuals (Vitalant Research Institute) via density-gradient separation according to institutional safety protocols. CD14+ monocytes were isolated from PBMC by magnetic negative selection and cultured at 37°C in 5% CO_2 on non-treated flat-bottom culture plates in 1X Iscove's Modified Dulbecco's Medium (IMDM, GIBCO 12440053), supplemented as follows. For dendritic cell differentiation: 1% Human AB Serum (Valley Biomedical HP1022HI), Penicillin-Streptomycin (100IU and 100μg/mL, respectively, Corning 30-002-CI), 1mM Sodium Pyruvate, 50ng/mL GM-CSF (Life Technologies PHC2015), 50ng/mL IL-4 (Life Technologies PHC0045); for macrophage differentiation: 20% Human AB Serum, Penicillin-Streptomycin, 1mM Sodium Pyruvate. Human THP1 cells were obtained from ATCC (ATCC TIB-202) and were cultured in RPMI 1640 with L-Glutamine (Corning MT10040CV), 10% FCS, 10mM HEPES (VWR 16777-02), 1mM sodium pyruvate (Fisher MT 30-0020-CI), and 0.5x Penicillin-Streptomycin. For macrophage-like cells, THP1s were stimulated with the 30nM PMA (Fisher BioReagents BP685-1) for 48 hours before harvest.

METHOD DETAILS

Primary human monocyte isolation and enrichment

Primary adult cells were obtained from consented healthy human donors from leukoreduction chamber residuals after Trima Accel apheresis (Vitalant) or, when sequencing consent was necessary, from STEMCELL, under a protocol approved by Vitalant or STEMCELL IRB. 50mL of human peripheral blood enriched in mononuclear cells was processed for each donor in accordance with institutional safety guidelines. Blood was diluted 1:1 with a 4mM EDTA PBS solution, then layered on top of Ficoll-Paque Plus density gradient medium (Sigma GE17-1440-03) in SepMate tubes (StemCell Technologies 85450). After centrifugation at 1200xg for 10 minutes at room temperature, blood separated into plasma, PBMC, granulocyte, and erythrocyte layers. The plasma was aspirated allowing the PBMC buffy coat to be decanted. It was then suspended in 2mM EDTA PBS and centrifuged at 400xg for 10 minutes at room temperature. PBMC were washed twice with 2mM EDTA PBS by centrifugation for 10 minutes at 300xg and then again at 200xg. Cells were counted, pelleted a final time at 200xg for 10 minutes, and resuspended at a concentration of 50×10^6 cells/mL in MACS buffer (PBS, 2mM EDTA, 0.5% BSA). At this point, 0.5×10^6 PBMC from each donor were removed for quality control flow cytometric analysis as described below. To enrich for CD14+ monocytes, 50μL/mL of CD14+ negative selection cocktail (StemCell 19359) was added and incubated for 5 minutes at room temperature. After incubation, suspensions were placed in an EasyEights EasySep Magnet (StemCell 18103) tube rack for 5 minutes. Enriched monocytes were carefully pipetted from the magnet, and 0.5×10^6 monocytes from each donor were again removed for flow cytometric quality control analysis as described below (See also Figures S1D–S1F).

Where noted, isolated PBMC or CD14+ monocytes were cryopreserved in FBS with 10% DMSO in a Mr. Frosty Freezing container (ThermoFisher 5100) stored at −80°C for 1-4 days before transfer to liquid nitrogen. Cryopreserved PBMC were carefully thawed and washed twice with MACS buffer before incubation with negative selection cocktail as described above.

Formation of Cas9-ribonucleoproteins

Cas9 ribonucleoproteins were produced as previously described (Hultquist et al., 2019). Briefly, lyophilized crRNA and tracrRNA (Dharmacon, see Key resources table, Oligonucleotides) were resuspended at a concentration of 160μM in 10mM Tris-HCL (7.4 pH) with 150mM KCl and immediately used or frozen at −80°C. No more than one thaw was allowed for any RNP reagent. Equal volumes of crRNA and tracrRNA were incubated at 37°C for 30 minutes to form an 80 μM guide RNA duplex; this was then incubated with an equal volume (2:1 RNA:Cas9 molar ratio) of 40μM Cas9 protein (UC Macrolab) at 37°C for 15 minutes to form RNPs at 20μM. RNPs were used immediately or stored at −80°C.

Nucleofection of Cas9-ribonucleoproteins into primary human monocytes

Isolated CD14+ monocytes were counted and 0.5-1 × 10^6 cells per nucleofection reaction were spun down at 200xg for 8 min. Supernatant was carefully and completely aspirated, and cells were resuspended in 20μL/reaction of room-temperature Lonza nucleofection buffer P2 (Lonza V4XP-2024). The cell suspension was gently mixed with 2.5μL/reaction of appropriate RNP and then pipetted into a 96-well-format nucleofection cuvette for the Lonza 4D X unit or Shuttle unit (Lonza). Except where explicitly stated, cassettes were nucleofected with code DK-100, immediately supplemented with 80μL pre-warmed culture medium, and rested in a dark, 37°C, 5% CO_2 incubator for 15-30 minutes. Subsequently cells were moved to a prepared non-treated, flat-bottom culture plate pre-filled with appropriate media for differentiation and subsequent analysis. One nucleofection reaction of 0.5×10^6 cells is sufficient to seed three wells of a 96-well plate or one well of a 48-well plate.

In vitro differentiation of monocyte-derived macrophages and dendritic cells

Cells were cultured in flat-bottom, non-treated cell culture plates in either 96-well (Corning 351172) or 48-well (Corning 351178) format. Twenty-four hours after nucleofection, after visually confirming cell adherence, the entire volume of media was exchanged for fresh, pre-warmed culture media. Three and five days after nucleofection, half of the culture media was removed and replaced with fresh media pre-warmed to 37°C. Media formulations are as follows. For MDDCs: 1X IMDM (GIBCO 12440053), 1% Human AB Serum (Valley Biomedical HP1022HI), Penicillin-Streptomycin (100IU and 100μg/mL, respectively, Corning 30-002-CI), 1mM Sodium Pyruvate, 50ng/mL GM-CSF (Life Technologies PHC2015), 50ng/mL IL-4 (Life Technologies PHC0045); for MDMs: 1X IMDM, 20% Human AB Serum, Penicillin-Streptomycin, 1mM Sodium Pyruvate.

Flow cytometric staining and analysis

To detach adherent MDMs and MDDCs for downstream flow cytometric analysis, cells were first spun for 5 minutes at 300xg, media was removed, and cells were incubated in Accutase Cell Detachment Solution (ThermoFisher Scientific 00-4555-56) for 15 minutes at 37°C. Cells were then transferred to V-bottom 96-well plates and resuspended in MACS buffer (PBS, 2mM EDTA, 0.1% Bovine Serum Albumin). Monocytes and PBMC did not require lifting.

To assess the quality of the CD14+ negative selection, samples before and after CD14 enrichment were stained with antibodies against CD14-PE (1:25)(Miltenyi 130-110-519) and CD16-APC (1:25)(Miltenyi 130-106-705).

For phenotypic analysis of MDMs and MDDCs, cells were blocked with Human TruStain FcX (Biolegend 422302) and stained at 4°C in a final volume of 50μL with the following antibodies: CD14-FITC (1:50)(Biolegend 301803), CD16-PE (1:50)(Biolegend 3G8), CD14-PE (1:25)(Miltenyi REA599), CD16-APC (1:25)(Miltenyi REA423), β2-Microglobulin-PE (1:100)(BD 551337), CD11b-BV650 (1:50)(BD 740566), CD11c-PerCP-Cy5.5 (BD 565227), CD206-BV421 (1:50)(BD 566281), LiveDead 510 (1:500)(Tonbo 13-0870-T100), HLA-DR-Pacific Blue (Life Technologies MHLDR28) and GhostDye Red 780 (1:500)(Tonbo 13-0865-T100). Compensation was performed using single-stained UltraComp eBeads Compensation Beads (Life Technologies 01-2222-42) and a mixture of live and killed MDMs or MDDCs for GhostDye Red 780 compensation. Samples were acquired on the Attune NxT Flow Cytometer (ThermoFisher) and analyzed using FlowJo software (FlowJo, LLC). For sample enrichment stains and gating strategy please see Figure S3C.

In vitro infection of MDMs by *Mycobacterium tuberculosis*

M. tuberculosis was grown to log phase in 7H9 liquid media (BD 271310) supplemented with Middlebrook OADC (Sigma M0678), 0.5% glycerol, 0.05% Tween-80 in roller bottles at 37°C. *M. tuberculosis* Erdman strain expressing eGFP under control of the MOP promoter was a gift from Dr. Sarah Stanley's laboratory. Macrophages were infected with fluorescent *M. tuberculosis* using a modified version of the spinfection protocol as previously described (Watson et al., 2015). Mycobacteria were washed in PBS three times and directly inoculated into the macrophage tissue culture wells at an MOI of 10. Following centrifugation, infected cells were incubated at 37°C for one day post-infection. The wells were washed with PBS and fixed with 4% PFA before staining for microscopy.

Quantification of *Mycobacterium tuberculosis* infection

After fixation in 4% PFA and transfer to PBS, plates were stored at 4°C for staining. Cells were permeabilized with 0.1% Triton X-100 (Sigma-Aldrich T8787) for 15 minutes, washed three times with 1X PBS and stained in a volume of 100 μL with CellMask Deep Red Stain (1:100,000) (ThermoFisher, H32721) for 30 minutes at room temperature in the dark. After staining, cells were washed three times with 1X PBS and stored in 100μL/well 1X PBS. Cells were then imaged using a Cellomics Arrayscan with a 10X objective (ThermoFisher) and analyzed using the HCS Studio quantitative analysis software (ThermoFisher) by defining cellular events based on the non-specific membrane Deep Red CellMask stain in the 650 channel and then quantifying infection by measuring mean fluorescent intensity in the 488nm channel.

HIV production

Macrophage-tropic HIV virus was generated using the HIV LAI-YU2 chimeric molecular clone, from the lab of Rahm Gummuluru. Virus plasmid (12μg) and 100μl PolyJet (Signagen) were diluted separately in two tubes of 625μL serum-free DMEM, then the two solutions were combined and vortexed to mix. After 15 minutes at room temperature, the transfection complexes were added to T175 flasks containing 293T/17 cells, which were gently rocked. Virus-containing culture supernatant was harvested 48 hours post transfection, spun at 400xg for 5 minutes and filtered through a 0.45μm filter. Virus was precipitated by addition of NaCl and 50% PEG-6000 to final concentrations of 300mM and 8.4%, respectively, followed by incubation for two hours at 4°C. Precipitated virus was pelleted for 40 minutes at 3500 rpm, resuspended in complete RPMI media at 50X concentration, frozen in aliquots on dry ice, and stored at −80°C. Virus was titered on wild-type MDMs prior to testing on knockout MDMs.

HIV infection, p24 staining and imaging

HIV LAI-YU2 was added to macrophages in 96-well format and incubated for 48 hours to allow for infection. After 48 hours, cells were washed twice in PBS (pH 7.4) and fixed in room temperature 4% formaldehyde for 30-60 minutes. Fixative was washed away with two PBS washes, with a final quench in PBS + 2% fetal calf serum (FCS, Invitrogen 26140079). Cells were permeabilized for 5 minutes with saponin buffer (PBS + 0.2% Saponin (Sigma S7900) + 2% FCS). Anti-p24 antibody (AIDS reagent 183-H12-5C) was diluted 1:500 in saponin buffer, 80 μL was added to each well, and the plate was incubated overnight at 4°C. Primary antibody was removed and

plates were washed 3 times with saponin buffer. Anti-mouse Alexa Fluor 488-conjuated antibody (Invitrogen A-21202) was diluted in saponin buffer (1:500), 80 μL was added to each well, and the plate was incubated for 2-3 hours at room temperature protected from light. Secondary antibody was removed, and the plate was washed twice with saponin buffer and once with PBS. Hoescht 33258 (Sigma 861405) was diluted into PBS for a final concentration of 1 μg/mL, and 80 μL was added to each well followed by a 5 minute incubation at room temperature. Hoescht buffer was removed and the plate was rinsed twice in PBS, then imaged on a CellInsight automated microscope using a 10X objective (ThermoFisher). Cells were identified using nuclear stain, enlarged cellular masks were drawn around the nuclear masks, and p24-positive cells identified by their high average fluorescence in the 488 channel.

Immunoblotting and protein quantification

To prepare protein samples, differentiated myeloid cells were harvested by aspirating the appropriate growth/differentiation media and then adding 100 μL of 2.5x reducing sample buffer (RSB, 1.872 mL 0.5M Tris-HCl pH 6.8, 6 mL 50% Glycerol, 3mL 10% SDS, 250 μL β-mercaptoethanol, 378 μL 1% bromophenol blue, 1X PBS) directly to each sample well. Cells were lysed by incubating for at least 3 minutes at room temperature, then lysates were transferred to 96-well PCR plates (USA Scientific 1402-9598). Plates were then heated at 95°C for 30 minutes and stored at −20°C. To prepare immunoblots, samples were thawed at room temperature, and 15 μL/lane was loaded into an 18-well 4%–20% Criterion TGX Gel (Bio-Rad 567-1094). Gels were run at 90 V for 30 minutes followed by 150 V for 50 minutes. The samples were then transferred at 0.25 A for 1 hour to a PVDF Membrane (Bio-Rad 1620177). Following protein transfer, membranes were blocked in 4% Milk PBST for 1 hour at room temperature, and then incubated in blocking solution overnight at 4°C with the following antibodies: rabbit monoclonal anti-ATP6V1A (1:1000)(Abcam EPR19270), rabbit polyclonal anti-GNE (1:1000)(Proteintech 25079-1-AP), rabbit polyclonal anti-SAMHD1 (1:1000)(Proteintech 12586-1-AP), rabbit monoclonal anti-β-actin (1:5000)(CST 4970P), mouse monoclonal α-GAPDH (1:5000)(Sigma G8795). Membranes were then washed three times in PBST for 5 minutes each, and then incubated with appropriate secondary antibody for 1 hour at room temperature. Membranes were washed an additional three times, then stained with Pierce ECL Western Blotting Substrate (ThermoFisher 32106). Exposures of the blots were taken with autoradiography film (Thomas Scientific XC59X) and developed with a medical film processor (Konica Minolta Medical & Graphic SRX-101A). Film was scanned at 300 pixels/inch and stored as 8-bit grayscale TIFF files. The level of protein expression for individual samples was quantified in FIJI (Schindelin et al., 2012) by inverting the images, subtracting the background, and determining the fluorescent intensity by measuring the integrated density of individual bands. The protein expression level was then reported as the relative fluorescence of the protein of interest with respect to the paired loading control.

Quantification of mutational efficiency by TIDE analysis

Cells were lysed in plate format in 50μL QuickExtract DNA Extraction Solution (Lucigen QE09050). Crude lysate was then incubated at 65°C for 20 minutes and 95°C for 20 minutes. Primers were designed with Primer3. PCR amplification was performed using Phusion 2X Master Mix HotStart Flex (New England Biolabs M0536L), 10μM primer pair (see Key resources table, Oligonucleotides), and approximately 100ng template DNA. PCR amplicons were subsequently sent for cleanup and Sanger sequencing. Mutational efficiency was then determined by comparison of non-targeting and gene-targeting sample chromatograms using the TIDE Web Tool (Brinkman et al., 2014).

Analysis of cell survival by luminescence

Relative cell viability was determined with CellTiter-Glo (Promega G7570) according to manufacturer's instructions. Briefly, fresh aliquots of CellTiter-Glo buffer and substrate were mixed and 100 μL of the resulting reagent was added to each well of a 96-well culture plate and the plate was put on a shaker for 2 minutes. Lysates were then moved to an opaque-walled 96-well plate (Costar 3912) and incubated at room temperature for 10 minutes. Luminescence was then recorded on an Enspire multimode plate reader (PerkinElmer).

RNA-seq analysis

Freshly isolated CD14 monocytes or day 7 MDM or MDDCs were lifted, pelleted, drained of supernatant and snap-frozen until processing. RNA isolation was conducted using the Qiacube RNeasy 96 QIAcube HT Kit (QIAGEN 74171) according to manufacturer's protocol. Gene expression profiling was carried out using a 3′ Tag-RNA-Seq protocol. Barcoded sequencing libraries were prepared using the QuantSeq FWD kit (Lexogen, Vienna, Austria) for multiplexed sequencing according to the recommendations of the manufacturer. The fragment size distribution of the libraries was verified via micro-capillary gel electrophoresis on a Bioanalyzer 2100 (Agilent, Santa Clara, CA). The libraries were quantified by fluorometry on a Qubit fluorometer (LifeTechnologies, Carlsbad, CA), and pooled in equimolar ratios. Libraries were sequenced on an Illumina NextSeq (Illumina, San Diego, CA). RNA isolation was conducted by the University of California, Davis (UC Davis) Real-Time PCR Research and Diagnostics Core and library preparation and sequencing were carried about by the UC Davis DNA Technologies and Expression Analysis Core.

RNA-Seq reads were processed with kallisto (version 0.44.0) using the *Homo sapiens* ENSEMBL GRCh38 (release 95) reference genome annotation. Transcript counts were aggregated at the gene level using the tximport (version 1.14.2) R package. Normalization, transformation, and principal components analysis were performed using the DESeq2 (version 1.26.0) R package. Gene set enrichment analysis was conducted with fgsea (version 1.16). All R code was run on version 3.6.3.

QUANTIFICATION AND STATISTICAL ANALYSIS

Flow cytometry data were analyzed using FlowJo software. Microscopy data were analyzed using HCS Studio quantitative analysis software. Immunoblots were analyzed with ImageJ. Data were visualized with GraphPad Prism or R with RStudio. Infection of *SAMHD1*-targeted MDMs was assessed by a repeated-measures one-way ANOVA followed by Dunnett's multiple comparisons test. All statistical details are present in relevant figure legends. RNaseq analysis was conducted in R with Rstudio.

Cell Reports, Volume 35

Supplemental information

Efficient generation of isogenic primary human myeloid cells using CRISPR-Cas9 ribonucleoproteins

Joseph Hiatt, Devin A. Cavero, Michael J. McGregor, Weihao Zheng, Jonathan M. Budzik, Theodore L. Roth, Kelsey M. Haas, David Wu, Ujjwal Rathore, Anke Meyer-Franke, Mohamed S. Bouzidi, Eric Shifrut, Youjin Lee, Vigneshwari Easwar Kumar, Eric V. Dang, David E. Gordon, Jason A. Wojcechowskyj, Judd F. Hultquist, Krystal A. Fontaine, Satish K. Pillai, Jeffery S. Cox, Joel D. Ernst, Nevan J. Krogan, and Alexander Marson

Figure S1

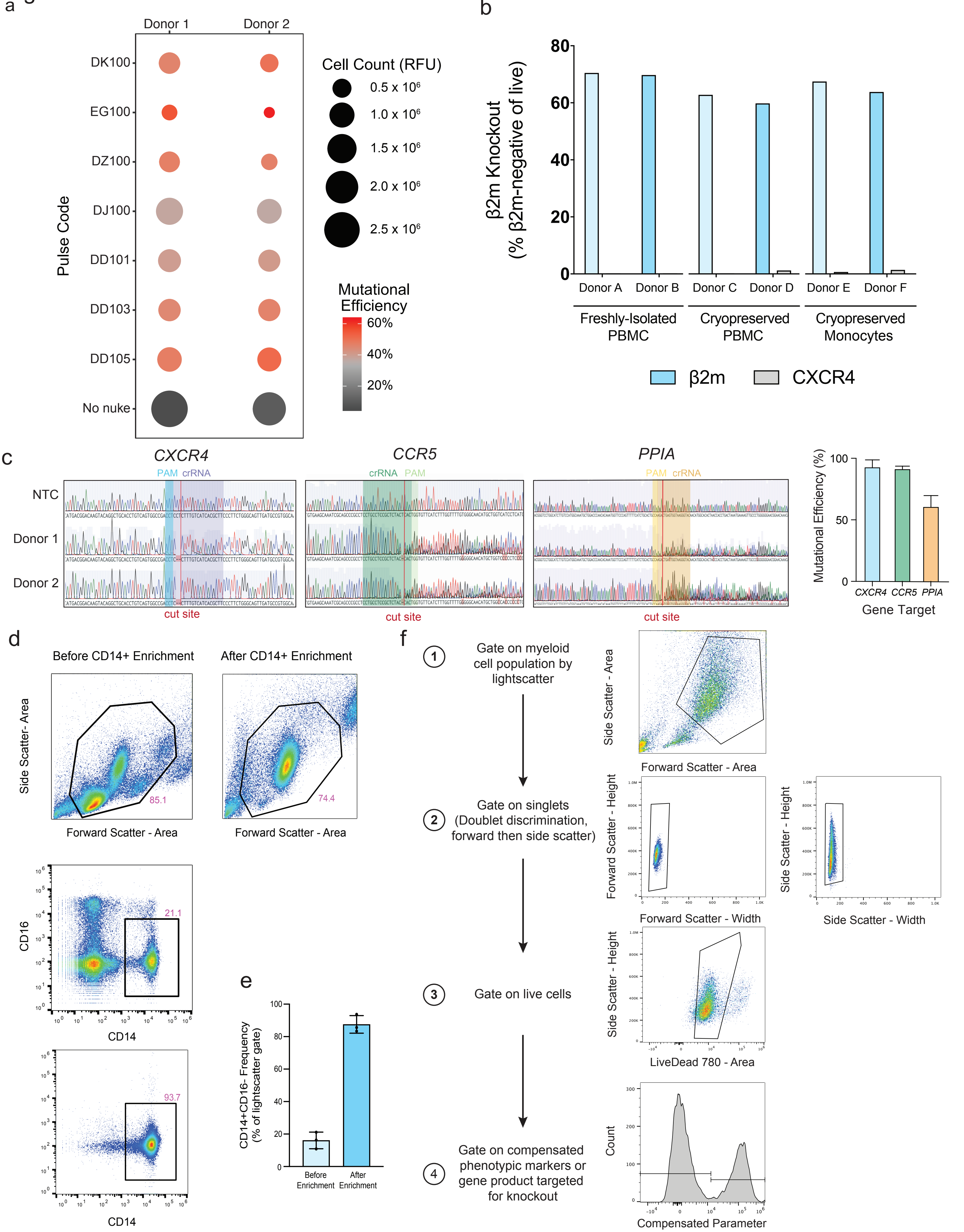

a
Donor 1
Donor 2
DK100
EG100
DZ100
DJ100
DD101
DD103
DD105
No nuke
Pulse Code
Cell Count (RFU)
0.5 x 10^6
1.0 x 10^6
1.5 x 10^6
2.0 x 10^6
2.5 x 10^6
Mutational Efficiency
60%
40%
20%
b
β2m Knockout (% β2m-negative of live)
Donor A
Donor B
Donor C
Donor D
Donor E
Donor F
Freshly-Isolated PBMC
Cryopreserved PBMC
Cryopreserved Monocytes
β2m
CXCR4
c
CXCR4
CCR5
PPIA
PAM crRNA
NTC
Donor 1
Donor 2
cut site
Mutational Efficiency (%)
Gene Target
d
Before CD14+ Enrichment
After CD14+ Enrichment
Side Scatter- Area
Forward Scatter - Area
85.1
74.4
CD16
CD14
21.1
93.7
e
CD14+CD16- Frequency (% of lightscatter gate)
Before Enrichment
After Enrichment
f
1 Gate on myeloid cell population by lightscatter
2 Gate on singlets (Doublet discrimination, forward then side scatter)
3 Gate on live cells
4 Gate on compensated phenotypic markers or gene product targeted for knockout
Side Scatter - Area
Forward Scatter - Area
Forward Scatter - Height
Forward Scatter - Width
Side Scatter - Height
Side Scatter - Width
LiveDead 780 - Area
Count
Compensated Parameter

Figure S1. Optimization of knockout in fresh and cryopreserved CD14+ monocytes and notes on isolation, Related to Figure 1 and STAR Methods. (a) Lonza nucleofection pulse code optimization of *SAMHD1* knockout using guide SAMHD1-2 in CD14+ monocytes freshly isolated from blood of two healthy donors. All nucleofections occurred in buffer P2. Color indicates mutational efficiency determined by TIDE, size of circles indicates relative surviving cell count measured by CellTiter-Glo fluorescent assay in relative fluorescence units (RFU), mean of technical triplicates. Cells were also visually monitored for health and morphology. **(b)** Knockout efficiency in CD14+ monocytes freshly isolated from blood (Donors A and B), CD14+ monocytes freshly isolated from cryopreserved PBMC (Donors C and D), and cryopreserved CD14+ monocytes (Donors E and F). Knockout at the targeted *β2m* locus was determined by TIDE compared to the non-targeting control (blue bars, left side of each donor); grey bars represent the TIDE knockout efficiency at the *β2m* locus of off-target *CXCR4* RNPs (grey bars, right side of each donor.) **(c)** Genomic analysis of knockout target sites allows for quantification of mutational efficiency independent of gene product expression. Left, representative chromatograms of non-targeting control (top) and edited (middle, Donor 1; bottom, Donor 2) sequences, with crRNA sequence, cut site and protospacer-adjacent motif (PAM) highlighted; right, quantification of editing efficiency by TIDE. Bars represent mean ± SD of two (*PPIA*) or four (*CXCR4, CCR5*) biological replicates. **(d)** Representative lightscatter (top) and CD14 vs. CD16 staining (bottom) of density-separated PBMC (left) and negatively selected CD14+ monocytes (right). **(e)** Quantification of enrichment by magnetic negative selection across three donors. Bars represent mean ± SD with individual points marked. **(f)** Panels illustrating four-step sample gating strategy for myeloid cells.

Figure S2

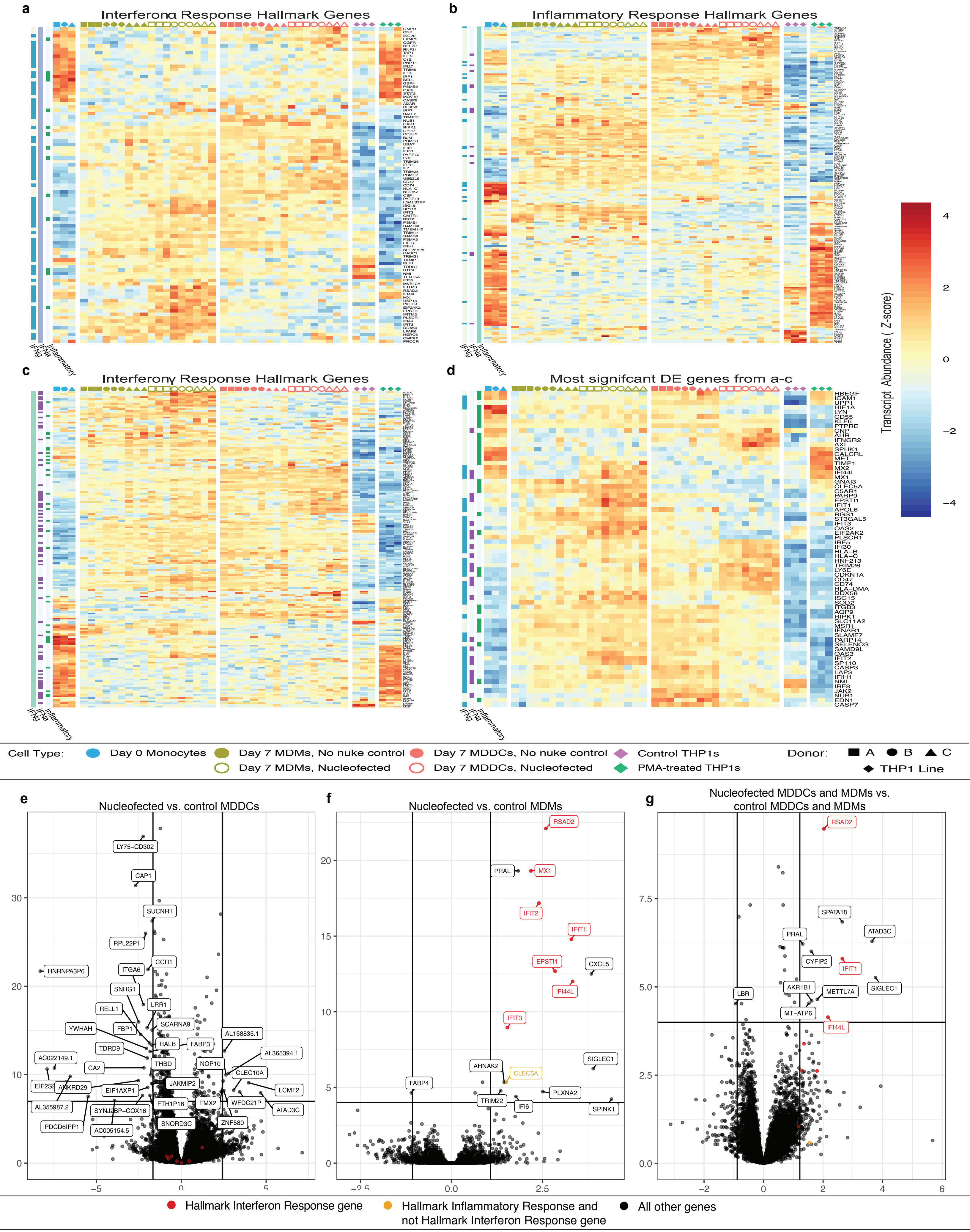

a
Interferonα Response Hallmark Genes
b
Inflammatory Response Hallmark Genes
c
Interferonγ Response Hallmark Genes
d
Most signifcant DE genes from a-c
IFNg
IFNa
Inflammatory
HBEGF
ICAM1
UPP1
HIF1A
LYN
CD55
KLF6
PTPRE
CNP
AHR
IFNGR2
AXL
SPHK1
CALCRL
MET
TIMP1
MX2
IFI44L
MX1
GNAI3
CLEC5A
C5AR1
PARP9
EPSTI1
IFIT1
APOL6
RGS1
ST3GAL5
IFIT3
OAS2
EIF2AK2
PLSCR1
IRF5
IFI30
HLA−B
HLA−C
RNF213
TRIM26
LY6E
CDKN1A
CD47
CD74
HLA−DMA
DDX58
ISG15
SOD2
ITGB3
AQP9
RIPK1
SLC11A2
MSR1
IFNAR1
SLAMF7
PARP14
SELENOS
SAMD9L
OAS3
IFIT2
SP110
CASP3
LAP3
IFIH1
NMI
IRF8
JAK2
NUB1
EDN1
CASP7
Transcript Abundance (Z-score)
4
2
0
−2
−4
Cell Type:
Day 0 Monocytes
Day 7 MDMs, No nuke control
Day 7 MDMs, Nucleofected
Day 7 MDDCs, No nuke control
Day 7 MDDCs, Nucleofected
Control THP1s
PMA-treated THP1s
Donor:
A
B
C
THP1 Line
e
Nucleofected vs. control MDDCs
LY75−CD302
CAP1
SUCNR1
RPL22P1
CCR1
ITGA6
HNRNPA3P6
SNHG1
LRR1
RELL1
SCARNA9
YWHAH
FBP1
AL158835.1
TDRD9
RALB
FABP3
AL365394.1
AC022149.1
CA2
THBD
NOP10
CLEC10A
EIF2S3
ANKRD29
EIF1AXP1
JAKMIP2
LCMT2
AL355987.2
SYNJ2BP−COX16
FTH1P16
EMX2
WFDC21P
ATAD3C
PDCD6IPP1
AC005154.5
SNORD3C
ZNF580
f
Nucleofected vs. control MDMs
RSAD2
PRAL
MX1
IFIT2
IFIT1
EPSTI1
CXCL5
IFI44L
IFIT3
SIGLEC1
AHNAK2
CLEC5A
FABP4
PLXNA2
TRIM22
IFI6
SPINK1
g
Nucleofected MDDCs and MDMs vs. control MDDCs and MDMs
RSAD2
SPATA18
ATAD3C
PRAL
CYFIP2
IFIT1
AKR1B1
METTL7A
SIGLEC1
LBR
MT−ATP6
IFI44L
Hallmark Interferon Response gene
Hallmark Inflammatory Response and not Hallmark Interferon Response gene
All other genes

Figure S2. Selected transcript abundance from monocytes, THP1 cells, and nucleofected and control MDMs and MDDCs, and differentially expressed transcripts, Related to Figure 2. (a-d) Relative transcript abundance for transcripts in the **(a)** Hallmark Interferon Alpha Response gene set**, (b)** Hallmark Interferon Gamma Response gene set or **(c)** Hallmark Inflammatory Response gene set (Liberzon et al., 2015). As the three sets are overlapping, the set memberships for each gene are displayed to the left of each heatmap. **(d)** Relative transcript abundance for leading edge genes from **(a-c)** with adjusted *P* value < 0.01 that drive differences between nucleofected and unperturbed MDMs or MDDCs. **(e-g)** Volcano plot of **(e)** nucleofected versus control day 7 MDDCs, **(f)** nucleofected versus control day 7 MDMs, **(g)** and combined nucleofected versus control day 6 MDDCs and MDMs. Transcripts in the top and bottom 1% by $\log_2$ fold change (x axis, cutoffs depicted by vertical lines) with adjusted *P* values less than 1×10^{-7} (panel **e**) or 1×10^{-4} (panels **f,g**) are labeled. Red dots indicate genes in the Hallmark Interferon Alpha Response or Hallmark Interferon Gamma Response gene sets (Liberzon et al., 2015); orange dots indicate genes not in the above two gene sets within the Hallmark Inflammatory Response gene set.

Figure S3

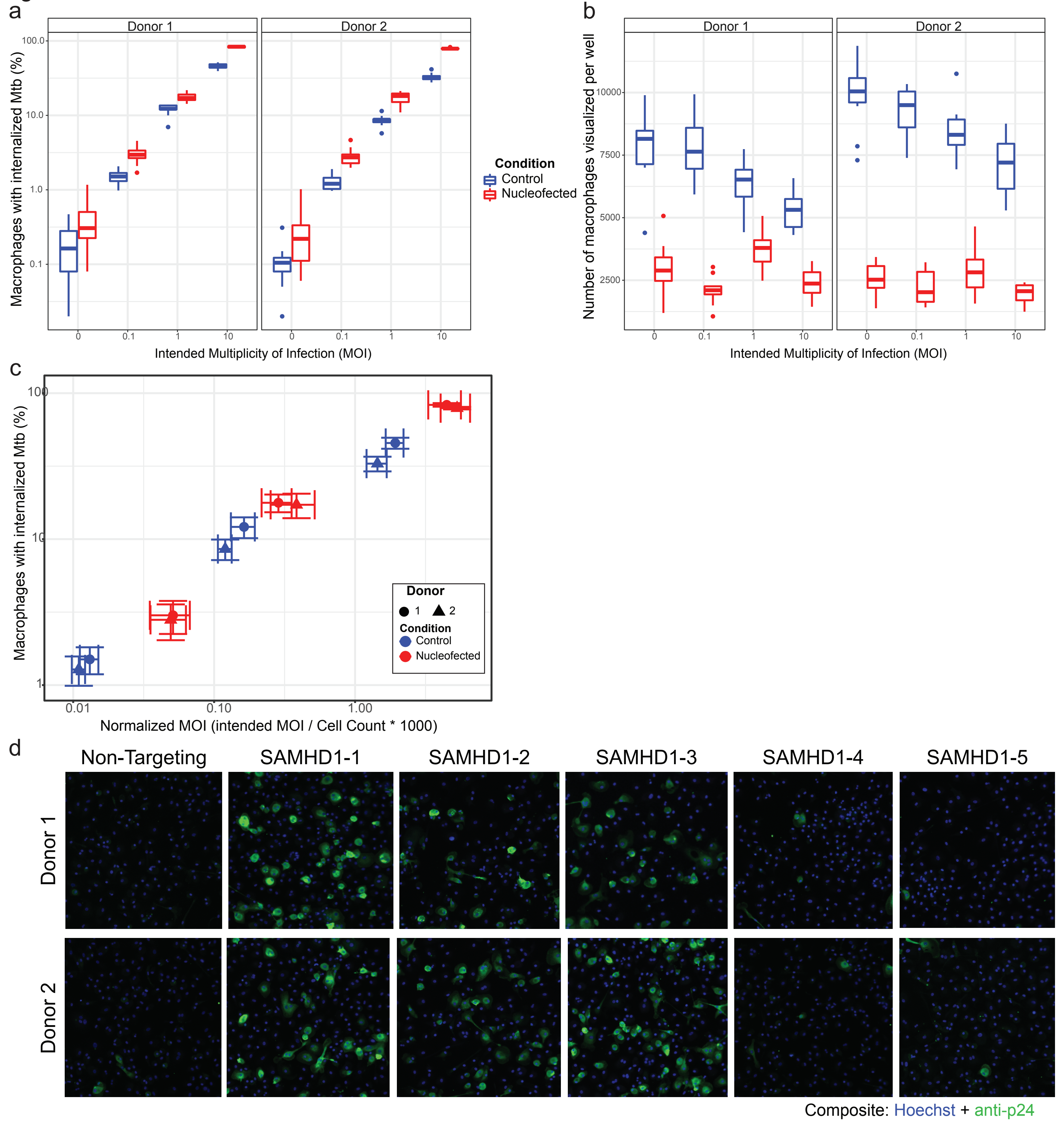

e

Dunnett's Multiple Comparison	Mean Difference (% infection)	95% CI of difference	Adjusted P Value
Non-Targeting vs. SAMHD1-1	21.05%	6.017% – 36.09%	0.0199
Non-Targeting vs. SAMHD1-2	20.55%	11.31% – 29.79%	0.0053
Non-Targeting vs. SAMHD1-3	19.08%	-0.5983% – 38.76%	0.0543
Non-Targeting vs. SAMHD1-4	1.10%	-0.4300% – 2.623%	0.1156
Non-Targeting vs. SAMHD1-5	0.82%	-0.5857% – 2.234%	0.1849

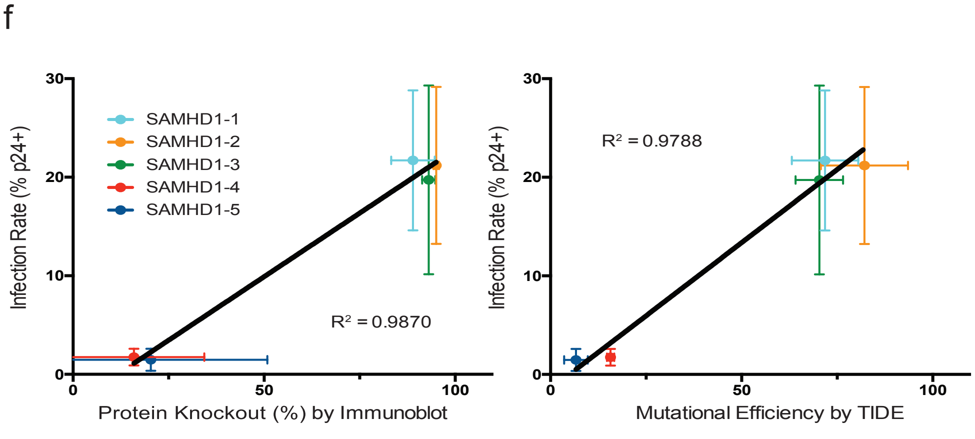

Figure S3. Quantification of phagocytosis of *Mycobacterium tuberculosis* and infection by HIV-1, Related to Figures 2-3.
(a-c) Quantification of phagocytosis of *Mycobacterium tuberculosis* (*Mtb*) by nucleofected and unperturbed day 7 monocyte-derived macrophages (MDMs). **(a)** Percentage of macrophages with internalized *Mtb* by intended multiplicity of infection (MOI). Box and whisker plots summarize 12 technical replicates, where the box depicts mean and interquartile range with max and min, excluding outliers, represented by the end of the whiskers. **(b)** Number of macrophages per well by intended multiplicity of infection (MOI). Box and whisker plots summarize 12 technical replicates, where the box depicts mean and interquartile range with max and min, excluding outliers, represented by the end of the whiskers. **(c)** Percentage of macrophages with internalized *Mtb* by normalized MOI (normalized MOI = intended MOI / cell count in thousands). Error bars represent standard deviation. **(d-f)** Knockout of the host restriction factor SAMHD1 leads to increases in HIV infection in a manner correlated with guide efficiency. **(d)** Representative composite images of HIV-1 infection from Donors 1 and 2 comparing cells nucleofected with RNPs made from each *SAMHD1*-targeting crRNA and cells nucleofected with control non-targeting RNPs. Blue, Hoechst; green, staining of the HIV-1 antigen p24. **(e)** Statistical comparison of infection rates (percent of cells staining positive for HIV-1 antigen p24). All values were generated by GraphPad Prism using a repeated measures one-way ANOVA followed by Dunnett's multiple comparison test. **(f)** Correlation of HIV-1 infection rate and knockout efficiency as measured by immunoblot (left panel) or mutational efficiency as measured by Sanger sequencing quantified by TIDE (right panel). Points represent mean across four donors, except for immunoblot of guide 1 (n = 2) and TIDE of guides 1, 3 and 4 (n = 3); error bars represent standard deviation. Line of best fit and R^2 values were generated by linear regression in GraphPad Prism.
